# SDGD-YOLO: a lightweight detection model for assessing the ripeness of black raspberries in complex greenhouse environments

**DOI:** 10.3389/fpls.2026.1843631

**Published:** 2026-05-15

**Authors:** Wenyan Zhao, Xue Ding, Jinliang Wang

**Affiliations:** 1 Faculty of Geography, Yunnan Normal University, Kunming, China; 2 Key Laboratory of Resources and Environmental Remote Sensing, Yunnan Provincial Universities, Kunming, China; 3 Yunnan Provincial Engineering Research Centre for Geospatial Information Technology, Kunming, China

**Keywords:** black raspberries, complex greenhouse environment, knowledge distillation, ripeness detection, Soft-NMS-GIoU, YOLO V13n

## Abstract

In complex greenhouse environments, black raspberries present challenges such as dense clusters, variable fruit sizes, continuous colour changes during ripening, and interference from foliage and uneven lighting. These issues lead to high false positives and false negatives in existing ripeness detection models, limiting their application in intelligent precision harvesting. To address this, this study proposes SDGD-YOLO, a novel ripeness detection model. First, a StarNet architecture is embedded into the feature extraction network to enhance cross-channel interaction and global semantic association, improving the extraction of key ripeness features. Second, a DySample dynamic upsampling module is introduced during multi-scale feature fusion to adapt to variations in fruit size and morphology across ripeness stages. Third, a collaborative optimization mechanism combining Soft-NMS and GIoU is applied in post-processing to reduce redundant bounding boxes and improve localization accuracy, effectively mitigating false positives and negatives under occlusion. Finally, a refined knowledge distillation mechanism via teacher-student joint training enhances detection performance while maintaining lightweight inference. The experimental results show that, compared with the baseline YOLOv13n, SDGD-YOLO achieves a precision of 91.0% (an increase of 3.6%), a recall of 71.8% (an increase of 4.7%), and an F1-score of 80.68% (an increase of 4.69%). with mAP50 and mAP50–95 reaching 81.7% (an increase of 5.7%) and 68.7% (an increase of 6.6%) respectively. Meanwhile, the number of model parameters has been reduced to 1.988 M, and GFLOPs to 5.8 G, achieving an effective balance between improved accuracy and reduced computational complexity. The SDGD-YOLO proposed in this study provides a technical reference for the accurate detection of berry ripeness in complex greenhouse environments.

## 1 Introduction

With the rapid growth of precision agriculture, computer vision-based automated sensing systems have emerged as a critical technological pillar pushing the change of orchard production toward intelligent and refined methods (Ghazal et al., 2024). These intelligent sensing systems provide a solid foundation for automated harvesting, fruit grading, and yield assessment by monitoring crop growth parameters in real time and analysing them precisely (Jeffrey and Mukesh, 2024). Blackberries are a typical high-value-added fruit since they are high in anthocyanins and antioxidants, have high edible and medicinal value, and are in great demand in the market (Yaqiong et al., 2024). The Yunnan Plateau, with its distinct climatic conditions—including abundant sunshine and significant diurnal temperature variations—and naturally favourable growing environment, has emerged as one of the primary production areas for blackberry cultivation; yield and quality have a direct impact on farmers’ economic income.

The nutritional content and flavour profile of black raspberries are directly determined by their maturity; it also impacts post-harvest shelf life and processing quality, which is critical to guaranteeing their high added value (Hayyat et al., 2025; Nisar et al., 2025). Furthermore, as a highly valuable but highly perishable aggregate fruit (Yaqiong et al., 2025), black raspberries have an exceptionally short harvesting window, putting strict constraints on harvest timing and accuracy. Traditional methods of assessing black raspberry ripeness rely heavily on manual visual grading, which is inefficient, has a high degree of subjective interpretation, significant errors in ripeness assessment, and high labour costs, making it difficult to meet the testing requirements of large-scale, facility-based cultivation (Chenglin et al., 2024b).

In recent years, with the rapid advancement of computer vision technology, the YOLO series of deep learning-based object detection algorithms has provided a new technical paradigm for crop phenotyping in orchard environments, gradually replacing traditional manual visual inspection methods (Wang and Kang, 2025). By employing high-resolution imaging equipment in conjunction with advanced vision algorithms, it is possible to capture subtle morphological and spectral characteristics of fruit (Zhujun et al., 2023; Anjali et al., 2024). This integrated approach has become the mainstream technical direction for crop ripeness detection (Jie et al., 2024). In research on the application of YOLO series models to crop ripeness detection, existing studies have largely focused on detection tasks involving fruits with larger sizes or distinct ripeness characteristics in open-field cultivation environments, such as apples (Liu et al., 2024), citrus fruits (Binbin et al., 2024), pears (Bonora et al., 2021) and mangoes (Koirala et al., 2019). For example, Sanchez et al. (2023) utilised CNN image processing to determine apple ripeness, achieving an accuracy rate of 86.84%. Hongxing, (2018) employed machine vision and Android mobile platform technology to conduct research on rapid, non-destructive detection of citrus ripeness, achieving an accuracy rate of 90.7%. For strawberry ripeness detection, the LS-YOLOv8s model, based on YOLOv8 and incorporating the LW-Swin Transformer, aims to address issues arising from complex growing environments, interference from light intensity, and occlusion caused by clustered strawberries, thereby enabling accurate detection and grading of ripe strawberries (Yang et al., 2023). However, there has been limited research on the ripeness detection of small-sized berries, such as blackberries, in complex greenhouse environments. Compared to large-fruited crops grown in open fields, complex greenhouse environments provide more stable and favourable growing conditions for blackberries; they also exacerbate the difficulty of ripeness detection—due to uneven light distribution, severe shading from branches and foliage, and dense overlapping of fruits. Furthermore, the small size of blackberry fruits and the subtle differences in colour and morphological characteristics across different stages of ripening present even more severe challenges for ripeness detection: Firstly, black raspberry fruits grow in dense clusters and are frequently obscured by foliage; combined with the effects of uneven lighting, the spectral reflectance characteristics of the fruits are prone to overlap with those of background shadows, dead leaves and other interfering objects, significantly increasing the rate of missed detections and reducing detection accuracy; Secondly, black raspberries at different stages of ripeness exhibit significant ‘intra-class similarity’, with extremely low feature distinguishability, which places extremely high demands on the feature extraction capabilities of detection models (Chenglin et al., 2024a). As the latest iteration in the YOLO series, YOLOv13n introduces a more efficient multi-stage feature extraction module into its backbone network compared to YOLOv8 and YOLOv11, and improves the cross-layer fusion paths within the feature pyramid. This significantly enhances both object detection accuracy and speed in complex scenarios whilst maintaining a compact parameter count, outperforming most existing object detection models in terms of both speed and accuracy. Based on the above analysis, this study addresses the core challenges and shortcomings of black raspberry ripeness detection in the complex greenhouse environment of the Yunnan Plateau. Using YOLOv13n (Mengqi et al., 2025) as the baseline model, this study undertakes a systematic co-design approach tailored to agricultural scenarios. We construct an improved YOLOv13n model that balances compactness with high accuracy, enabling precise and rapid detection of blackberry ripeness in complex greenhouse environments. This provides new insights for research into the intelligent detection of blackberry ripeness in high-altitude facilities under complex lighting conditions. Unlike traditional incremental module stacking, this study constructs a closed-loop optimisation pathway across four dimensions: backbone feature enhancement, multi-scale fusion adaptation, post-processing robustness optimisation, and model lightweighting while preserving fidelity. This ensures that each module functionally supports the others in a progressive, layered manner, collectively addressing the complex challenges in blackberry detection. Specifically, the core contributions and design logic of this paper are as follows:

1. To address the issue of feature confusion caused by the continuous colour transition of ripe blackberries and their similarity to the background foliage, the lightweight feature extraction network StarNet (Xu et al., 2024) was introduced to replace the original backbone network. During the ripening process, blackberries exhibit a continuous hue transition from greenish-blue to pink to purplish-black; furthermore, under certain lighting conditions, the deep purple of ripe fruit and the greenhouse foliage background tend to exhibit similar grey-scale values and blurred edges. By enhancing cross-channel feature interaction and spatial detail retention, StarNet more effectively preserves fine-grained visual features—such as fruit texture, hue, and edge patterns—whilst maintaining the model’s lightweight nature. This enhances the model’s ability to distinguish subtle differences in ripeness, thereby laying a stable foundation for subsequent multi-scale feature fusion.
2. To address the issues of inconsistent multi-scale feature representation and missed detection of small objects caused by the wide variation in fruit size, the Dynamic Sampling (DySample) (Wenze et al., 2023) module is deployed within the neck network structure to replace the traditional sampling module. Traditional sampling employs a fixed interpolation strategy, which struggles to adapt to the drastic changes in fruit size across the different stages of blackberry ripening. DySample can adaptively adjust sampling positions and weights based on feature distribution, thereby more accurately recovering key information from high-resolution feature maps. It is particularly suitable for complex greenhouse scenarios characterised by severe occlusion from branches and leaves and frequent fruit overlap. This module forms a collaborative ‘feature extraction–feature reconstruction’ chain with the aforementioned StarNet backbone; the former ensures feature quality, whilst the latter optimises scale adaptation, jointly enhancing the consistency of fruit detection across different stages of ripeness.
3. To address the issues of candidate box overlap and localisation errors caused by the dense clustering structure of blackberries (with individual clusters typically bearing 5–12 fruits), the Soft-NMS-GIOU (Navaneeth et al., 2017) algorithm was applied in the post-processing stage to replace the traditional NMS algorithm. This approach incorporates distance and scale information whilst accounting for the overlapping relationships between detection boxes, applying a continuous attenuation rather than a strict rejection to candidate boxes. Consequently, black raspberry ripeness targets that are spatially close but semantically distinct are retained. This effectively mitigates the issue of false suppression in areas where black raspberry fruits are clustered, improving the completeness and stability of black raspberry detection results, and thereby enhancing the overall recall rate. This module works in concert with the aforementioned DySample: DySample ensures the accurate reconstruction of multi-scale features, whilst Soft-NMS-GIoU safeguards target integrity in dense scenes; together, they reduce both false negatives and false positives in densely clustered regions.
4. To retain the performance improvements of the aforementioned modules under lightweight limitations, knowledge distillation (Geoffrey et al., 2015) is used to improve detection performance while not considerably increasing model complexity. For the multi-stage black raspberry ripeness detection task, this improves the model’s ability to recognise samples at the ripeness stage boundaries. While maintaining lightweight and real-time capabilities, it significantly improves detection accuracy and recall, as well as the model’s generalisation ability in complicated greenhouse situations. This mechanism functions as a ‘fidelity enhancer’ for the preceding modules, guaranteeing that model performance is successfully increased while remaining lightweight and real-time, resulting in a virtuous loop of ‘increasing accuracy and preserved lightweight design’.

In summary, the SDGD-YOLO model proposed in this study establishes a progressive collaborative optimisation pathway to address the three core challenges—feature confusion, scale invariance, and dense occlusion—associated with black raspberry ripeness detection in the complex greenhouse environment of the Yunnan Plateau; the various modules support one another functionally and are seamlessly integrated. Experimental results demonstrate that this model achieves accurate and rapid detection of black raspberry ripeness within the complex greenhouse environment of the Yunnan Plateau. It provides high-confidence fruit coordinates and ripeness grading information to support harvesting decisions, thereby effectively reducing post-harvest losses caused by misjudgements of ripeness. The model’s effective balance between compactness and accuracy gives it the potential for transfer to practical application scenarios such as intelligent inspection equipment and portable detection terminals. These research findings provide technical support for the precise control of ripeness in protected blackberry cultivation. They also explore a viable technical pathway for the standardisation and intelligent development of the plateau’s distinctive berry industry, offering practical value in reducing manual harvesting costs and post-harvest losses.

## 2 Test data

### 2.1 Image acquisition and data annotation.

**Figure 1:**
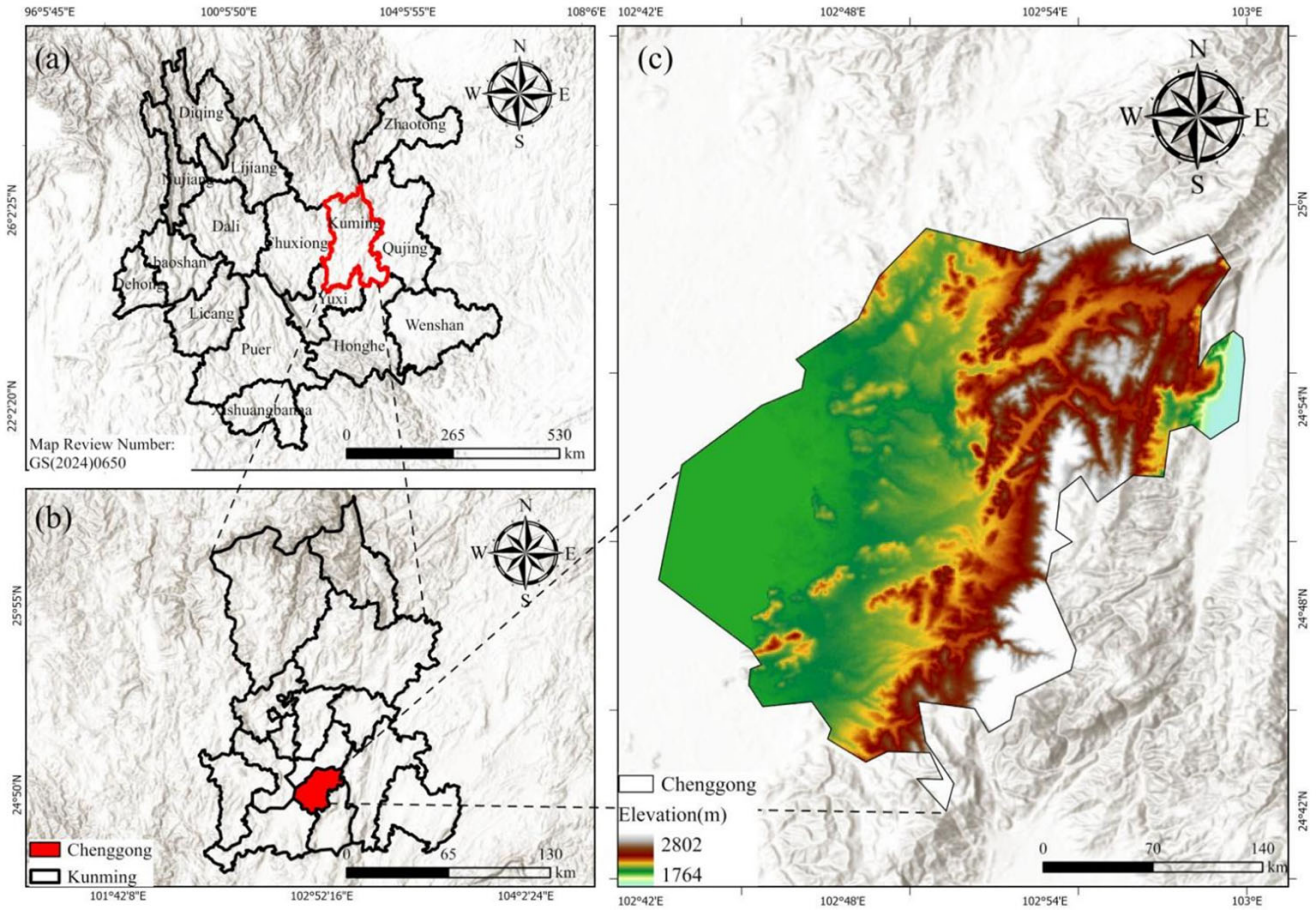
Research area overview map.

The black raspberry samples for this investigation were obtained from a greenhouse in the experimental orchard at Wanxichong Town, Chenggong District, Kunming City, Yunnan Province (102°83′ E, 24°88′ N, 1,950 m above sea level), as indicated in **Figure 1**. The greenhouse is a conventional multi-span polytunnel, and samples were taken from four rows of black raspberry plants. To capture the varying degrees of ripeness during the autumn fruiting season of black raspberries and ensure that the dataset reflects the entire developmental process of the fruit from green-ripe to full maturity, data were collected on three separate occasions: September 22, October 1, and October 12, 2025. These three collecting rounds coincided to diverse weather conditions and stages of fruit growth, thereby enhancing the dataset’s diversity and providing a representative sample base that was closely connected with local growing conditions for future model training.

**Figure 2:**
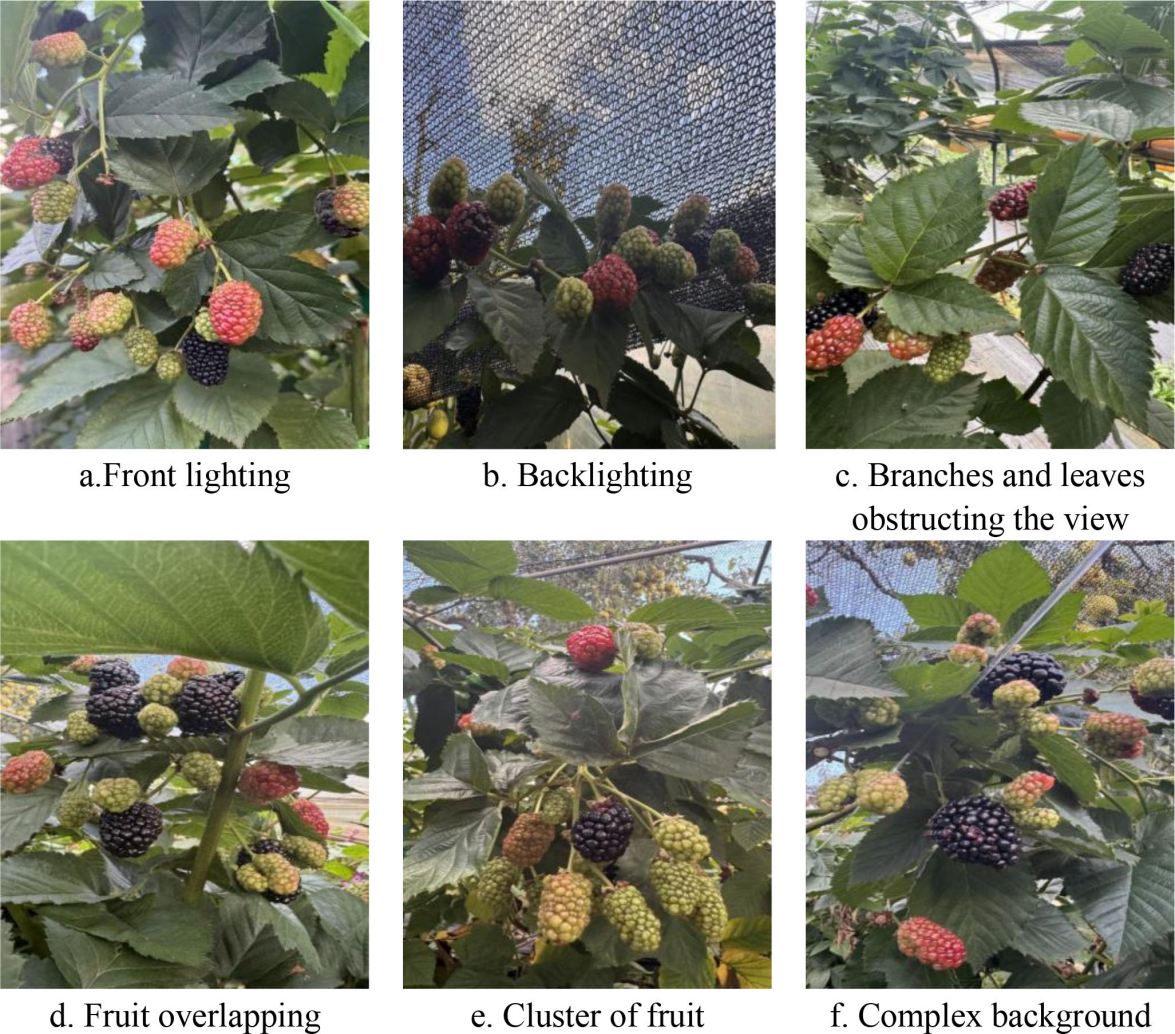
Partial image data of black raspberry fruit.

This study used a mainstream smartphone, specifically the iPhone 15 Pro, to collect images. This device was chosen since it is commonly used by farmers in black raspberry growing areas, ensuring widespread applicability. Furthermore, using a common mobile device for data gathering minimises the need for specialised imaging equipment, increasing the practical usefulness of the detection model proposed in this study. The image resolution was consistently fixed at 4284 × 5712 pixels. During shooting, the system automatically changed the camera’s shutter speed based on ambient light conditions; the flash was turned off to prevent localised overexposure; and white balance was set to automatic mode to ensure correct colour reproduction. The handheld gadget was held at the same height as the blackberry clusters, about 0.5-1.2 m away from the fruit, to imitate producers’ viewing angles during routine inspection duties. All photos were stored in JPG format to ensure that the parameters obtained were consistent and standardised. To ensure the dataset’s diversity and representativeness, as well as to improve the model’s generalisation performance, the collected samples cover a wide range of lighting conditions, levels of foliage obstruction, fruit overlap states, and interference scenarios within complex greenhouse environments. A total of 1,832 raw blackberry pictures were captured. After data collection was completed, photographs that were extremely blurred, out of focus owing to camera shake, featured too few black raspberries, or had the main subject stretching beyond the frame were removed, as were comparable duplicate images. Following the filtering process, 1,738 good raw photos were retrieved; a partial dataset is depicted in **Figure 2**.

To precisely establish maturity grades and ensure annotation quality in order to meet model training requirements, all images were detailed annotated using the Roboflow online annotation tool. The annotation procedure precisely followed the standards for classifying raspberry fruit maturity as outlined in the national standard ‘Specifications for Raspberries’ (GB/T 27657-2011). Black raspberry maturity was graded into four levels based on their real growing features in the Yunnan Plateau greenhouse environment and commercial harvesting criteria. The specific classification requirements are listed below as shown in **Table 1**.

**Table 1:** Criteria for classifying the ripeness of blackberries.

Maturity grade	Percentage of coloured area	Fruit appearance characteristics	Corresponding national standard description
Immature	0%~20%	Fruit is greenish-blue, surface is dull, flesh is firm, and fruit is small	Unripe
Turning	20%~60%	Fruit is pink to light purple, colouring spreads from the tip towards the stem, and the surface becomes glossy	Early maturity
Mature	60%~95%	Fruit is deep purple, with a high gloss on the surface; flesh is soft and suitable for commercial harvesting	Mature
Overmature	>95%	Fruit is purple-black, with reduced surface gloss; flesh is excessively soft and some fruit shows signs of shrivelling	Overripe

The proportion of the coloured region is computed by visually estimating the projected surface area of the fruit, which is estimated based on the entire visible area of the fruit. Where a portion of the fruit is concealed, the evaluation is based exclusively on the visible region.

**Figure 3:**
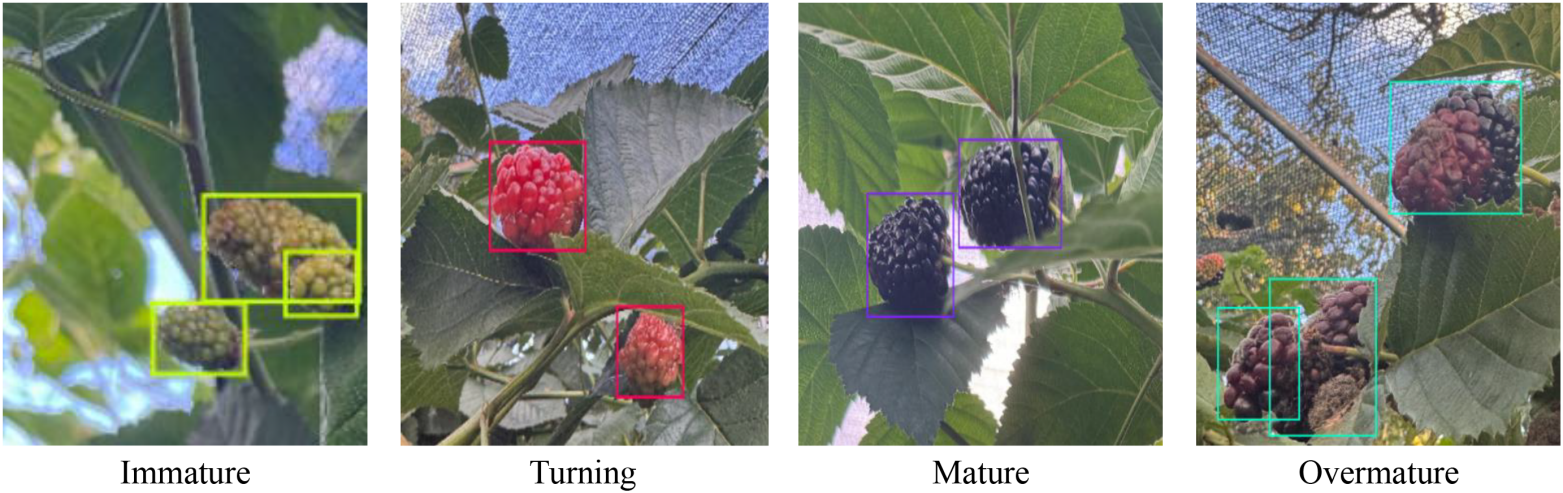
Data set annotation diagram.

As illustrated in **Figure 3**, black raspberry fruits were classified into four maturation stages: green, colour-changing, ripe, and overripe. The author carried out the annotating procedure separately. Before annotation, the author thoroughly researched the GB/T 27657–2011 standard and, with on-site assistance from the agronomist at the experimental orchard, carried out physical comparisons and picture sample verification of the typical characteristics of the four ripeness phases. For samples where there was disagreement, an agronomist from the experimental orchard was invited to help with the determination. The annotated files are saved in the normal XML format. To guarantee appropriate training, parameter tuning, and performance validation of the detection model, the annotated photos were divided into training, validation, and test sets in a 7:2:1 ratio, with 1,155, 381, and 202 images each. Stratified sampling was used during the splitting procedure to verify that the distribution of maturity categories across the three subsets was consistent with the original dataset. This method substantially reduced evaluation bias, ensuring the reliability and reproducibility of model performance evaluations.

### 2.2 Data augmentation

**Figure 4:**
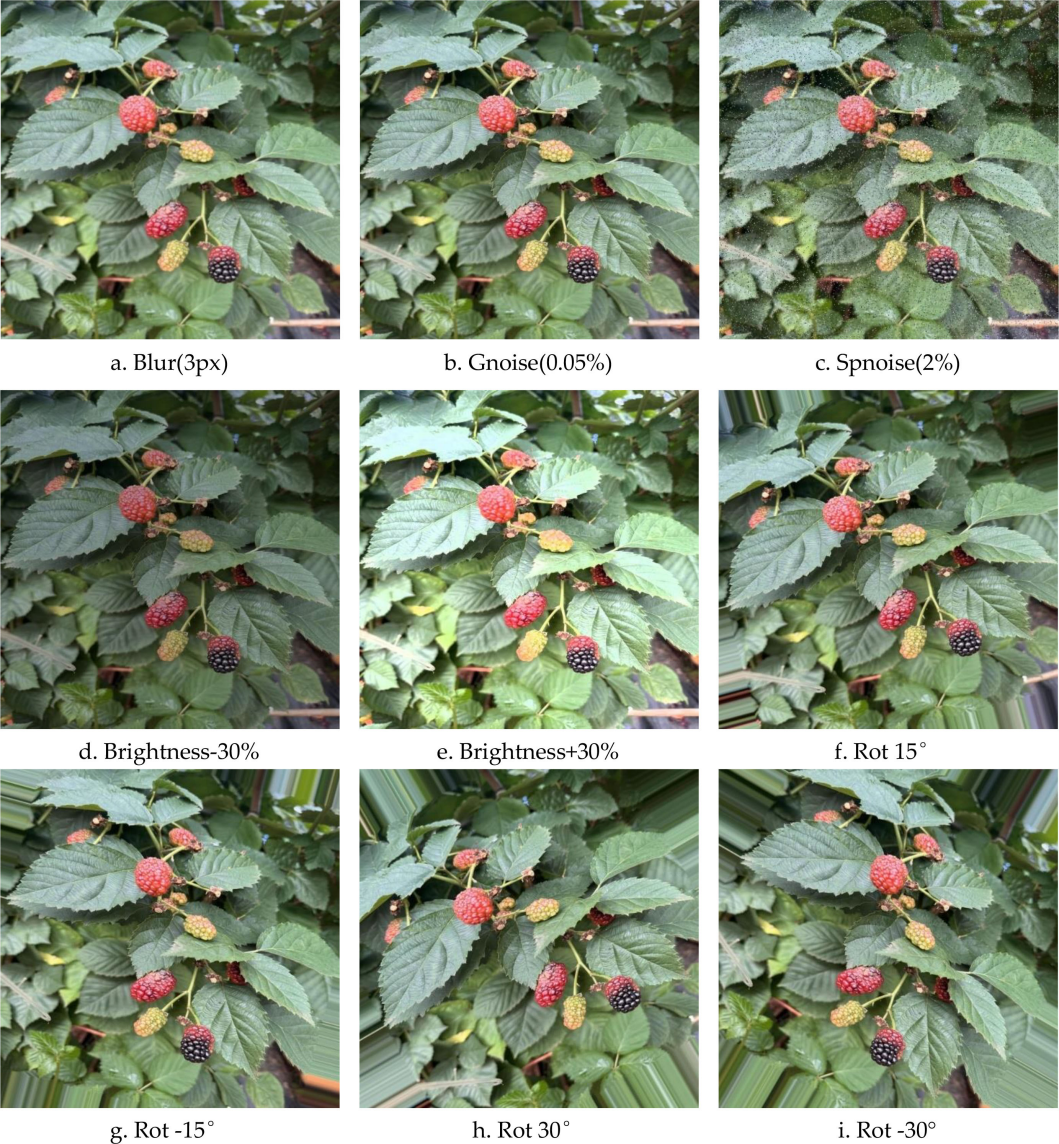
Schematic diagram of data augmentation methods.

The raw dataset acquired from a complicated greenhouse setting has an uneven distribution of image samples, with the ‘Mature’ and ‘Overmature’ categories under-represented, sparse, and class-imbalanced. To improve the detection model’s ability to recognise these two categories, as well as its generalisation and robustness in complex greenhouse environments, this study employs a targeted, controlled data augmentation strategy, with augmentation processing limited to these two under-represented categories. As indicated in **Figure 4**, five particular enhancing strategies were used: Enhancements include Gaussian blur, Gaussian noise, salt-and-pepper noise at ±15° and ±30°, small-angle rotation, and brightness modification by ±30%. Non-position-transformation enhancements reuse the original annotation information directly, whereas rotation-based enhancements correct the target bounding box coordinates at the same time to ensure annotation information consistency and data annotation quality. The five enhancement strategies chosen for this study are designed to emulate real-world interference scenarios that could arise in complicated greenhouse systems.

To prevent the risk of data distribution skew or overfitting due to excessive enhancement, this study independently determines application probability thresholds for each type of enhancement approach, allowing for exact control over the level of enhancement. Specifically, the application probability thresholds for Gaussian blur, Gaussian noise, salt-and-pepper noise, rotational rotation, and brightness adjustment are 30%, 20%, 10%, 40%, and 30%, respectively. To avoid data homogenisation caused by fixed enhancement patterns, a random sampling method is utilised to assess if a single sample from a minority class should be subjected to the appropriate enhancement technique. That is, each sample’s decision to apply each sort of improvement is decided independently depending on the set probability, rather than using a predefined combination of enhancements. It should be noted that the data augmentation techniques used in this study were only performed after the dataset was split. The original photos were separated into training, validation, and test sets in a 7:2:1 ratio, and data augmentation was applied to the images representing the’mature’ and ‘overripe’ stages within each of these three sets. This sequence of actions assures that there are no’similar samples’ from augmentation between the training and validation sets, or between the training and test sets, effectively eliminating data leaking. Following data augmentation, the original dataset was increased from 1,738 to 3,557 photos, effectively addressing class imbalance and enriching sample variety. The extended dataset consists of 2,489 training images, 711 validation images, and 357 testing images.

## 3 Online system for assessing the ripeness of blackberries

### 3.1 SDGD-YOLO network model

**Figure 5:**
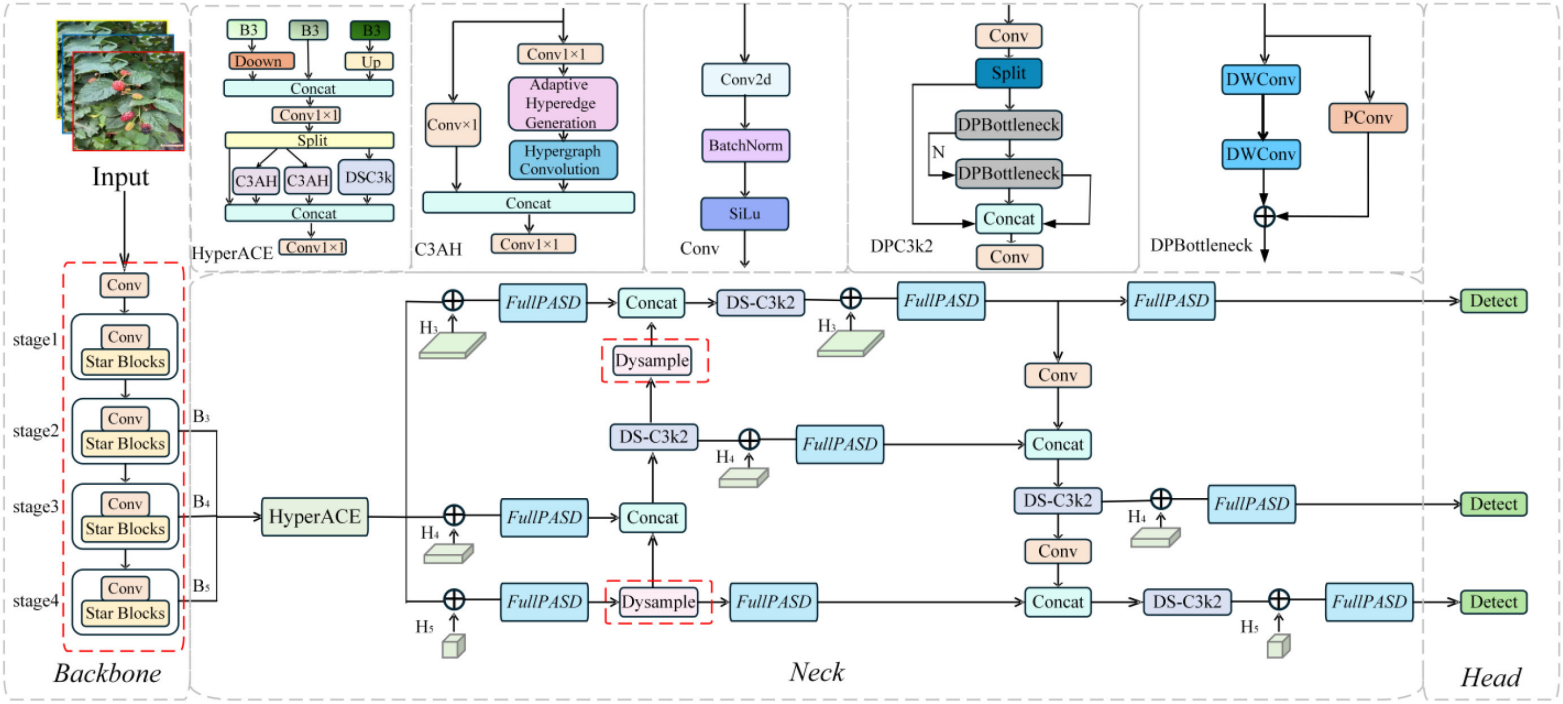
SDGD-YOLO network model architecture.

The SDGD-YOLO network model is based on the YOLOv13n framework and incorporates targeted improvements to the network architecture and training strategy whilst retaining the original end-to-end detection advantages. The structure of the SDGD-YOLO network model is shown in **Figure 5**. Firstly, in the feature extraction stage, the original YOLOv13n backbone network has been replaced with the lightweight feature extraction network StarNet, in order to enhance the model’s ability to capture fine-grained features of fruit whilst reducing the number of parameters and computational complexity. Second, in the neck network design, a dynamic sampling module (DynamicSampling, DySample) has been added to replace the typical fixed up-sampling procedure. This tries to overcome the issue of small-scale feature information being readily lost during multi-scale feature reconstruction, thus improving feature fusion across scales. Following that, during the detection findings’ post-processing stage, the Soft-NMS-DIOU technique is used to replace the original non-maximum suppression approach. This reduces the number of false negatives induced by harsh threshold suppression in instances of dense fruit distribution and local occlusion, boosting detection recall. Finally, a knowledge distillation approach was implemented during the model training phase. Establishing a knowledge transfer method between the teacher and student models improved detection accuracy and overall generalisation performance without significantly increasing model complexity. Through these multiple improvements, the model’s capacity to detect blackberries at various stages of ripeness in complicated orchard situations has considerably improved.

#### 3.1.1 StarNet

**Figure 6:**
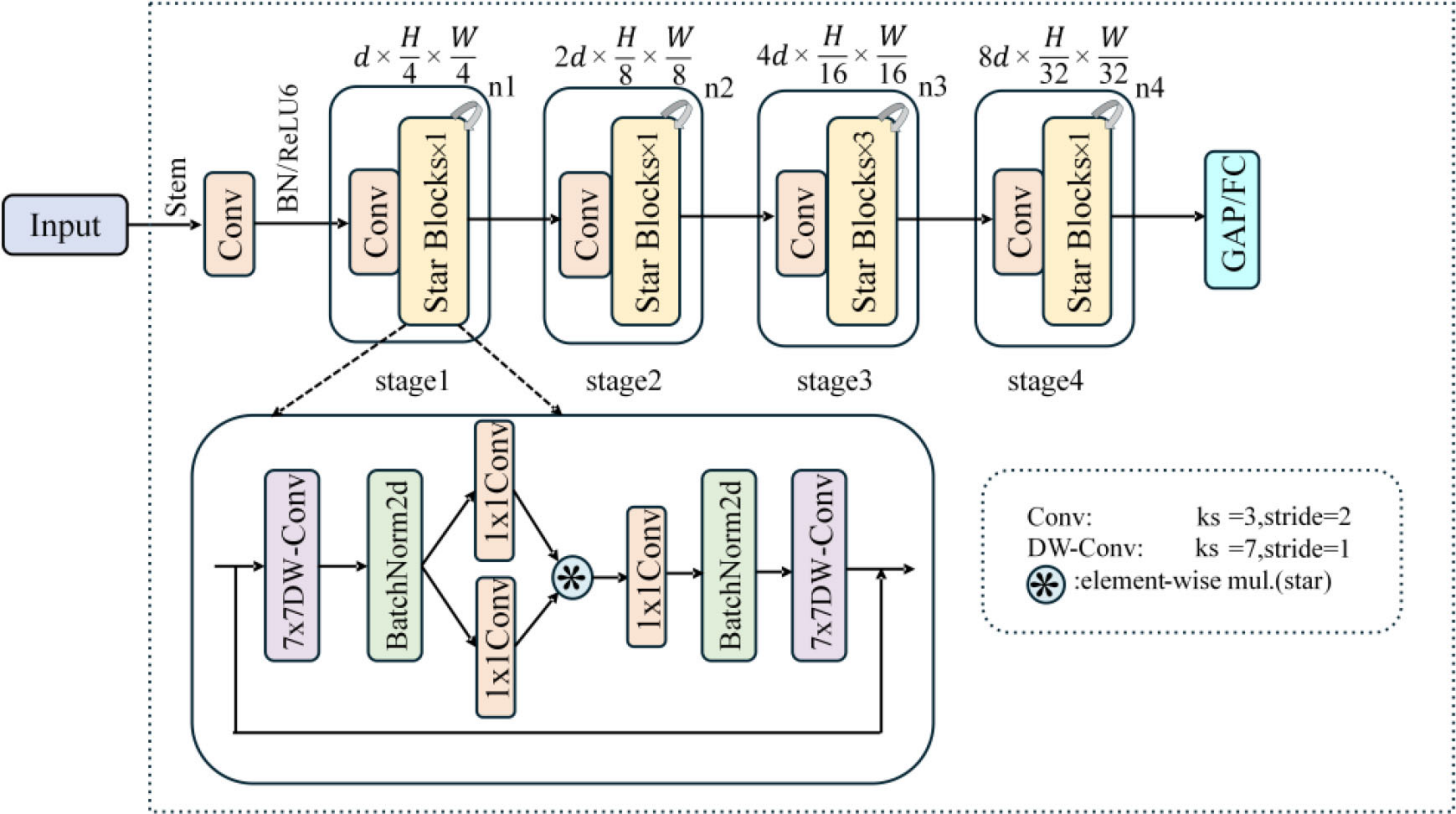
StarNet network architecture diagram.

The original YOLOv13n uses multi-layer conventional convolutions to extract image information. However, due to the small size of the fruit targets and the limited differences in colour and texture across different stages of ripeness, combined with complex backgrounds such as uneven lighting and occlusion by branches and leaves, traditional convolutional networks struggle to extract discriminative features while ensuring real-time performance. This frequently results in significant computing overhead, making them unsuitable for real-time detection tasks. Consequently, this study uses the lightweight convolutional neural network StarNet to replace the backbone network of YOLOv13n. **Figure 6** illustrates the network’s phased hierarchical design. At each level, the input features are initially downsampled using convolutional layers to minimise the size of the feature maps and extract preliminary information, followed by deeper feature extraction using several Star Block modules. At the network’s conclusion, Global Average Pooling (GAP) and Fully Connected (FC) layers collect and map multi-scale features, allowing for efficient global feature integration (Jiquan et al., 2019).

Star Block, the foundation of StarNet, extracts spatial features from an input feature map X using 7×7 depthwise convolution (DW-Conv). This procedure greatly decreases computing complexity while preserving important structural information. The features are subsequently treated to batch normalisation (BN) in order to stabilise the network training process. The features are linearly transformed, and channel expanded using two concurrent 1×1 convolutional branches. One branch uses a ReLU6 activation function after convolution to improve non-linear representation capacity. The outputs from the two branches are then blended using element-wise multiplication, which models the interactions between characteristics across multiple channels and captures higher-order correlations among them. The fused features are processed successively through 1x1 convolutions, batch normalisation, and 7x7 deep convolutions to integrate features and model contextual information. Finally, they are combined with the original input features via a residual link to mitigate the vanishing gradient problem and improve network stability, resulting in the final feature map.

The computational procedure unfolds as follows. Let *X* ∈ *R*^d^+1 denote the input feature vector, with *W_1_*and *W_2_* representing the weight matrices, and *B_1_*and *B_2_* the respective bias terms. A single layer of the star operation may thus be formulated as ( $W_{1}^{T}X+B_{1}$)*( $W_{2}^{T}X+B_{2}$), In simplified analysis, considering only the case without bias, this can be expanded as Equation (1):

(1) $$\begin{array}{c} W_{1}^{T}X*W_{2}^{T}X=(\sum_{i=1}^{d+1}w_{1}^{i}x^{i})(\sum_{j=1}^{d+1}w_{2}^{j}x^{j}) \end{array}$$

Further elaboration yields:

(2) $$\begin{array}{c} \sum_{i=1}^{d+1}\sum_{j=1}^{d+1}w_{1}^{i}w_{2}^{j}x^{i}x^{j}=\sum_{i=j}\alpha (i,j)x^{i}x^{j} \end{array}$$

Here, α(i,j) denotes the coefficient determined by the weight matrix, while $x^{i}$ represents the i-th component of the input feature vector As evident from Equation 2, the star operation is equivalent to performing second-order feature combination on the input features, thereby achieving dimensionality expansion in the implicit feature space. Through the stacking of numerous Star Blocks, the star movement can be recursively stated as:

(3) $$\left\{ \begin{array}{c} \begin{array}{l} S_{1}=\sum_{i=1}^{d+1}\sum_{j=1}^{d+1}w_{\left(1,1\right)}^{i}w_{\left(1,2\right)}^{i}x^{i}x^{j}\\ S_{2}=w_{2,1}^{T}S_{1}*w_{2,2}^{T}S_{1}\\ \ldots \\ S_{n}=w_{n,1}^{T}S_{n-1}*w_{n,2}^{T}S_{n-1} \end{array} \end{array}\right.$$

As demonstrated by Equation 3, after stacking n layers of Star Blocks, features are mapped to a high-dimensional latent space with a dimension approximately equal to $R^{d^{2^{n}}}$. This enables the network to possess formidable nonlinear expressive capabilities while maintaining an extremely low parameter count. In practical implementation, to satisfy detection accuracy requirements while further reducing model complexity, StarNet-s050 is selected as the backbone network for YOLOv13n. This network comprises four feature extraction stages, with the number of Star Blocks per stage being 1, 1, 3, and 1, respectively. A 3×3 convolutional layer is positioned at the start of each stage for downsampling and channel adjustment. The features collected are then processed through the Star Block module, which allows for in-depth feature extraction while remaining computationally lightweight. This process enables the generation of discriminative, multi-scale feature representations essential for accurate ripeness detection.

#### 3.1.2 Dysample

Traditional upsampling methods, such as nearest-neighbour interpolation and bilinear interpolation, struggle to effectively distinguish fruit targets from complex background features in complex orchard environments because they rely on fixed receptive fields and local neighbourhood information. Multi-scale feature fusion approaches have a tendency to smooth down and remove fine-grained fruit characteristics. This is especially evident in situations involving leaf occlusion, fruit overlap, and uneven lighting, where small fruits and ripening targets in the colour-changing phase are more likely to be obscured by background interference, resulting in a significant decrease in detection accuracy and recall rates. Furthermore, typical sampling approaches are extremely sensitive to noise and are prone to misclassifying leaf textures or high-luminance backgrounds as fruit characteristics, further compromising the model’s robustness.

To solve the aforementioned difficulties, this article incorporates the dynamic upsampling operator DySample into YOLOv13n’s neck network, re-modelling the upsampling process using a point-based sampling approach. Unlike standard approaches that use fixed interpolation kernels, DySample learns to produce sampling points, resulting in adaptive reconstruction of upsampled features. This effectively eliminates complicated background noise while minimising parameter redundancy and optimising computational resource usage (Yang and Qiu, 2024).

**Figure 7:**
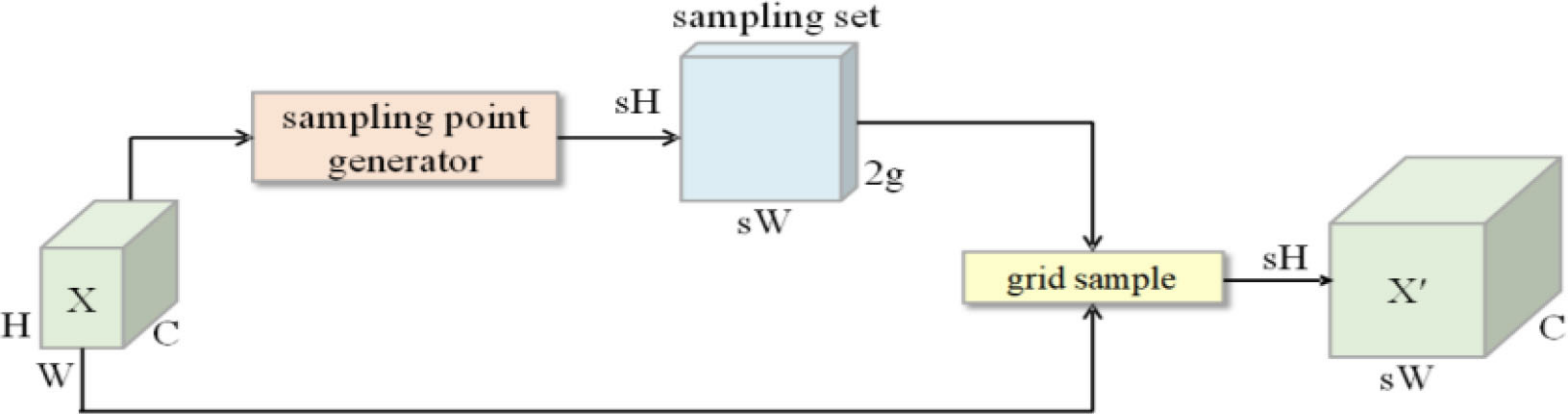
Dysample sampling process.

As depicted in **Figure 7**, DySample operates in two main phases: generating sampling points and resampling features. Initially, for a given input feature map *X*, the sampling point generator produces a set *S*. The network then utilises grid_sample to resample the input features, producing the upsampled feature map *X’*. This technique is formally expressed as shown in Equation (4):

(4) $$\begin{array}{c} X^{\prime }=grid\_sample(X,S) \end{array}$$

The set of samples *S* is acquired by combining the reference rule sampling network *G* with the computed offset *O*. The computational process is outlined as follows, as shown in Equation (5):

(5) $$\begin{array}{c} S=G+O \end{array}$$

**Figure 8:**
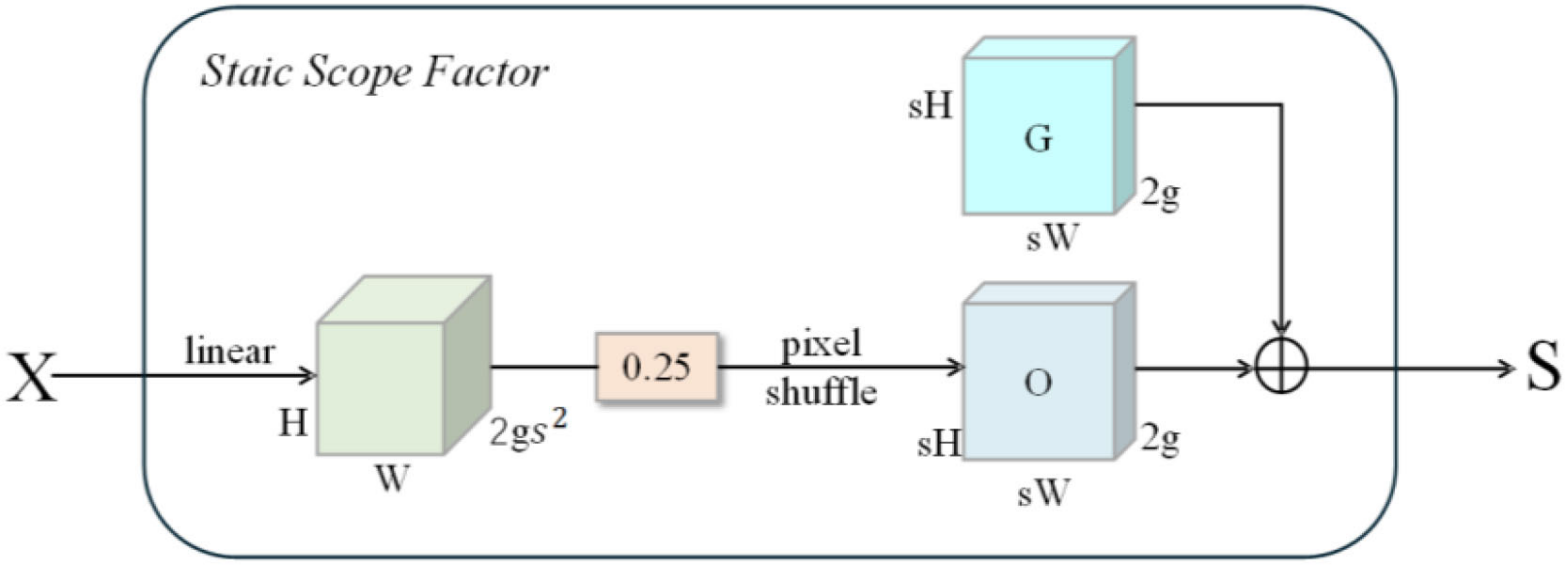
Structure of the sampling point generator.

**Figure 8** illustrates the structure of the sampling point generator constructed based on the static range factor. The generation of offset *O* employs a ‘linear mapping + pixel rearrangement’ approach. Given an oversampling factor, an input characteristic map of size *C×H×W* is first passed through a linear layer with input dimensionality *C* and output dimensionality *2s²*, yielding an offset feature of size *2s²×H×W*. Subsequently, a pixel reordering operation reshapes this into a *2×sH×sW* configuration, where 2 denotes the spatial displacement amount in both the *x* and *y* directions. The resulting sampling offset distribution aligns with the spatiotemporal scale of the upsampled feature map.

Under static range factor constraints, the generation of offsets may be expressed as follows, as shown in Equation (6):

(6) $$\begin{array}{c} O=0.25(linear(x)) \end{array}$$

Specifically, 0.25 denotes the static range scaling factor, which limits the maximum displacement of sample locations. This static range limitation technique efficiently prevents excessive displacement or geographical overlap of sampling locations, ensuring that the sampling distribution remains stable and reasonable. As a result, during the multi-scale feature fusion step, it better preserves the spatial structure and maturity classification features of black raspberries, increasing the model’s detection accuracy and robustness in complex orchard settings.

#### 3.1.3 Soft-NMS-GIoU

The traditional Non-Maximum Suppression (NMS) algorithm (Alexander and Van, 2006) typically employs hard suppression of candidate detection boxes based on an IoU threshold during the post-processing stage of object detection; that is, candidate boxes whose overlap with high-confidence detection boxes exceeds the threshold are removed directly. However, in the black raspberry ripeness detection job, due to the dense distribution of fruits, mutual occlusion, and considerable scale changes, hard threshold suppression is prone to generating the erroneous removal of real targets, leading to missed detection issues. The Soft-NMS algorithm used in this study effectively addresses the aforementioned concerns by gradually decreasing the confidence scores of detection boxes rather than removing them entirely. This algorithm’s basic workflow is similar to that of NMS, but it uses specific evaluation metrics for suppression windows. Equation 7 describes the evaluation concept for the NMS method, while Equation 8 describes the Soft-NMS algorithm:

(7) $$\begin{array}{c} s_{i}=\{\begin{array}{ll} s_{i}, & iou(M,b_{i})\leq N_{t}\\ 0, & iou(M,b_{i})\geq N_{t} \end{array} \end{array}$$

(8) $$\begin{array}{c} s_{i}=\{\begin{array}{ll} s_{i}, & iou(M,b_{i})\leq N_{t}\\ s_{i}(1-iou(M,b_{i})), & iou(M,b_{i})\geq N_{t} \end{array} \end{array}$$

In the formula, *i* denotes the category label; $\;s_{i}$ represents the score of the current detection box; $N_{t}$ indicates the IoU threshold; *M* is the detection box with the highest score; $b_{i}$ represents the corresponding initial detection box.

To solve the issue of discontinuous output results in Equation 8, this study uses an exponential decay function to gradually alter the confidence score based on the degree of overlap between detection boxes, avoiding the instability associated with threshold selection. The greater the degree of overlap, the more extreme the decay in the detection box’s confidence score; nonetheless, the candidate box is always kept for future ranking. The definition is as follows, as shown in Equation (9):

(9) $$\begin{array}{c} s_{i}=s_{i}e^{-\frac{ka{\left(M,b_{i}\right)}^{2}}{\sigma }},\forall b_{i}\notin 𝒟, \end{array}$$

In the formula, e denotes the natural logarithm; D denotes the set containing the final elements.

However, the overlap measure commonly employed in classic Soft-NMS is IoU, which just shows the proportion of overlapping area between detection boxes without considering their spatial relationship. When two detection boxes have no overlap or only a small overlap but are spatially close, IoU struggles to accurately capture their similarity, especially in scenarios involving densely clustered objects or small targets, which can easily result in insufficient or false suppression, as shown in Equation 10. To improve the suppression effect, this study incorporates the Generalised Intersection over Union (GIoU) (Zhaohui et al., 2020) as a measure of overlap within Soft-NMS’s Gaussian attenuation framework, resulting in the Soft-NMS-GIoU approach. By introducing a minimum bounding box constraint based on IoU, GIoU can provide an effective spatial similarity metric even when detection boxes do not overlap or overlap only slightly, thereby more comprehensively reflecting geometric relationships between detection boxes, as shown in Equation 11.

(10) $$\begin{array}{c} \text{IoU=}\frac{\left|{\text{B}}_{\text{gt}}\cap {\text{B}}_{\text{pre}}\right|}{\left|{\text{B}}_{\text{gt}}\cup {\text{B}}_{\text{pre}}\right|} \end{array}$$

(11) $$\text{GIoU}=\text{IoU}-\frac{\left|C-(B_{gt}\cup B_{pre})\right|}{\left|C\right|}$$

Where ${\text{B}}_{\text{gt}}$ denotes the True Bounding box, ${\text{B}}_{\text{pre}}$ denotes the Predicted Bounding box,and ∩ and ∪ denote the intersection and union, respectively. C represents the minimum bounding rectangle, which is the smallest rectangle fully encompassing both the ground truth bounding box ${\text{B}}_{\text{gt}}$ and the predicted bounding box ${\text{ B}}_{\text{pre}}$. |C| denotes the area of *C*.

In Soft-NMS-GIoU, the confidence score $S_{i}$ of the candidate detection box $b_{i}$ is updated according to a Gaussian decay function, calculated as follows, as shown in Equation (12):

(12) $$\begin{array}{c} S_{i}\leftarrow S_{i\;\cdot }\;exp(-\frac{GIoU{\left(M,b_{i}\right)}^{2}}{\sigma }) \end{array}$$

Here, *M* denotes the detected bounding box with the current highest confidence score, $GIoU(M,b_{i})$ represents the Generalised Intersection over Union score between detection box M and candidate box $b_{i}$, and σ is the Gaussian decay coefficient used to control the magnitude of confidence decay. Soft-NMS-GIoU enables more accurate differentiation between spatially proximate yet differently scaled detection boxes while preserving object integrity, thereby effectively reducing false suppression in densely populated scenes.

Building on Soft-NMS’s low false-negative rate, Soft-NMS-GIoU improves the capacity to distinguish between densely overlapping fruits and small targets. It significantly decreases false suppression due to occlusion, overlap, and scale fluctuations in complicated orchard situations, increasing overall detection accuracy and recall rate in blackberry ripeness detection tasks.

#### 3.1.4 Knowledge distillation

Hinton et al. initially proposed knowledge distillation (KD). Its central idea is to use a more powerful teacher model to transfer knowledge to a lightweight student model, allowing the student model to perform similarly to the teacher model despite limitations on parameter count and computational complexity. Depending on the source of the distilled information, distillation methods can be broadly classified as logical distillation (based on the output layer) or feature distillation (based on intermediate features) (Longrong et al., 2023). The former employs the output layer logits of the teacher and student models as the fundamental distillation objectives; by restricting the consistency of their class probability distributions, it directs the student model to learn the teacher model’s discriminative knowledge. The latter, on the other hand, uses the feature representations in the teacher and student models’ intermediate layers as distillation targets; by constraining the consistency of their feature space distributions, it allows the student model to learn the teacher model’s richer feature representation patterns (Borui et al., 2022). In the objective of recognising the ripeness of blackberries in complicated orchard environments, single-type distillation approaches frequently fail to extract all usable knowledge from the instructor model. Combining logical distillation and feature distillation provides complementary benefits: logical distillation ensures that the student model learns the discriminative knowledge of the teacher model, improving the accuracy of class predictions; feature distillation, on the other hand, improves the student model’s feature representation capabilities, increasing the robustness of target localisation and feature discrimination (Li et al., 2023).

**Figure 9:**
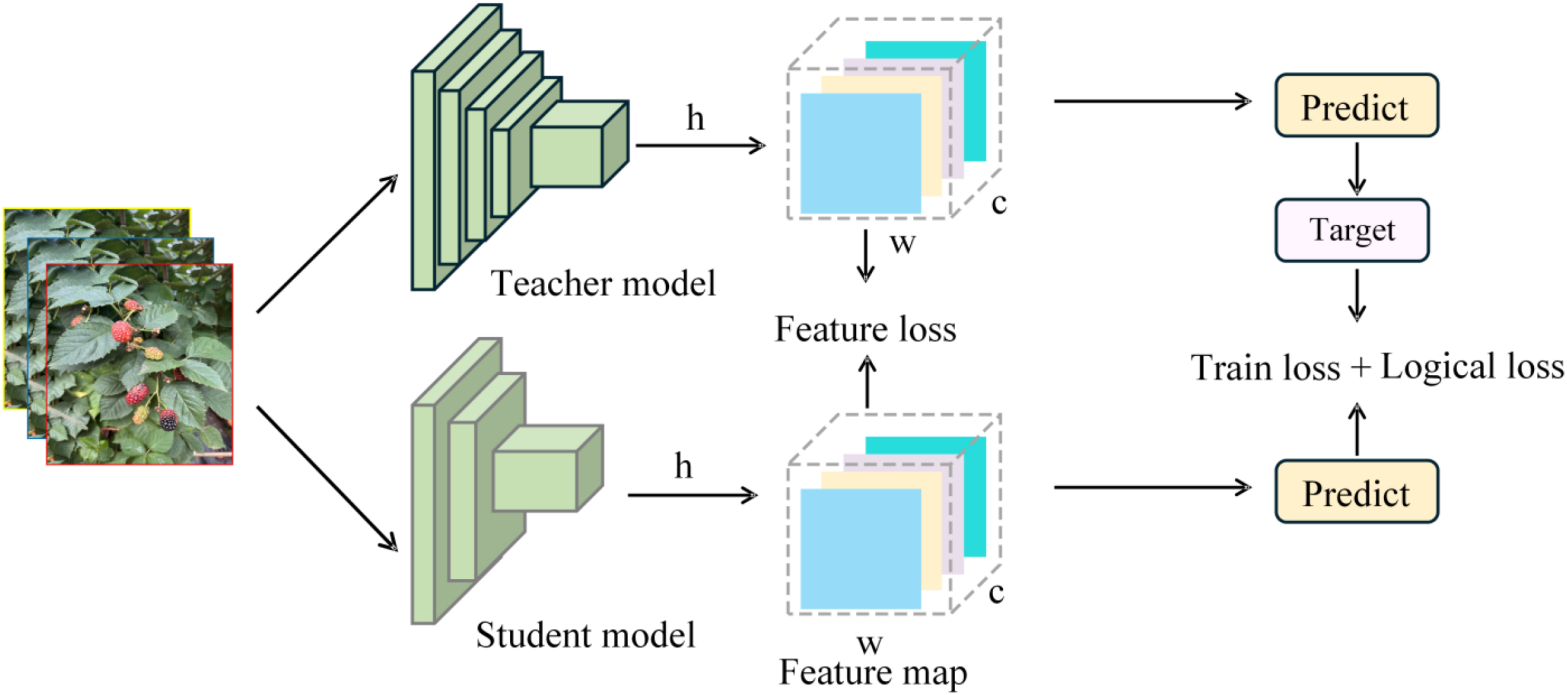
Schematic diagram of knowledge distillation.

To address the problem of low accuracy in lightweight object detection models in complex orchard environments, this work used an approach that combined BCKD-based logical distillation with channel-level knowledge. **Figure 9** shows a distillation scheme. This method was utilised to compensate for the performance of the modified YOLOv13n model, increasing the accuracy and robustness of blackberry ripeness detection.

BCKD (Block-Correlation Knowledge Distillation, BCKD) (Wang et al., 2023) is an improved method of logical distillation that effectively addresses the distillation challenges caused by inconsistent feature map sizes or differences in channel dimensions by introducing a multi-layer perceptron (MLP) and a pre-trained classifier from the teacher model, thereby broadening the applicability of logical distillation. By aligning the class prediction distributions at the detection head’s output layer between the teacher and student models, the student model is guided to learn the teacher model’s discriminative knowledge. The class-based distillation loss is defined as follows Equation (13):

(13) $$\begin{array}{c} L_{BCKD}=\sum_{i=1}^{N}\text{KL}(p_{t}(x_{i})\|p_{s}(x_{i}) \end{array}$$

Here, $p_{t}$ and $p_{s}$ are the category probability distributions of the teacher and student models, respectively, and *N* represents the sample size. By reducing this loss, the student model’s category predictions gradually converge with those of the teacher model.

CWD (channel-wise knowledge distillation) (Changyong et al., 2021) is a channel-wise feature distillation technique. It initially normalises each channel’s feature responses into probability maps before constraining the differences between the teacher and student models’ probability maps for the associated channels using the Kullback-Leibler (KL) divergence. By limiting the KL divergence between the probability maps of the teacher and student networks for the corresponding channels, the student model aligns with the teacher model’s regions of significant response across each channel during training, improving feature representation capabilities. The loss function is defined as follows (Equation 14):

(14) $$\begin{array}{c} L_{CWD}=\sum_{c=1}^{C}\sum_{i=1}^{W\cdot H}\phi (y_{c}^{t},i)\ln\frac{\phi (y_{c}^{t},i)}{\phi (y_{c}^{s},i)} \end{array}$$

Here, $y_{c}^{t}$ and $y_{c}^{s}$ denote the feature responses of the teacher model and student model, respectively on channel *c*, *W* and *H* represent the spatial dimensions of the feature map, and ϕ(.) denotes the normalisation function.

**Figure 10:**
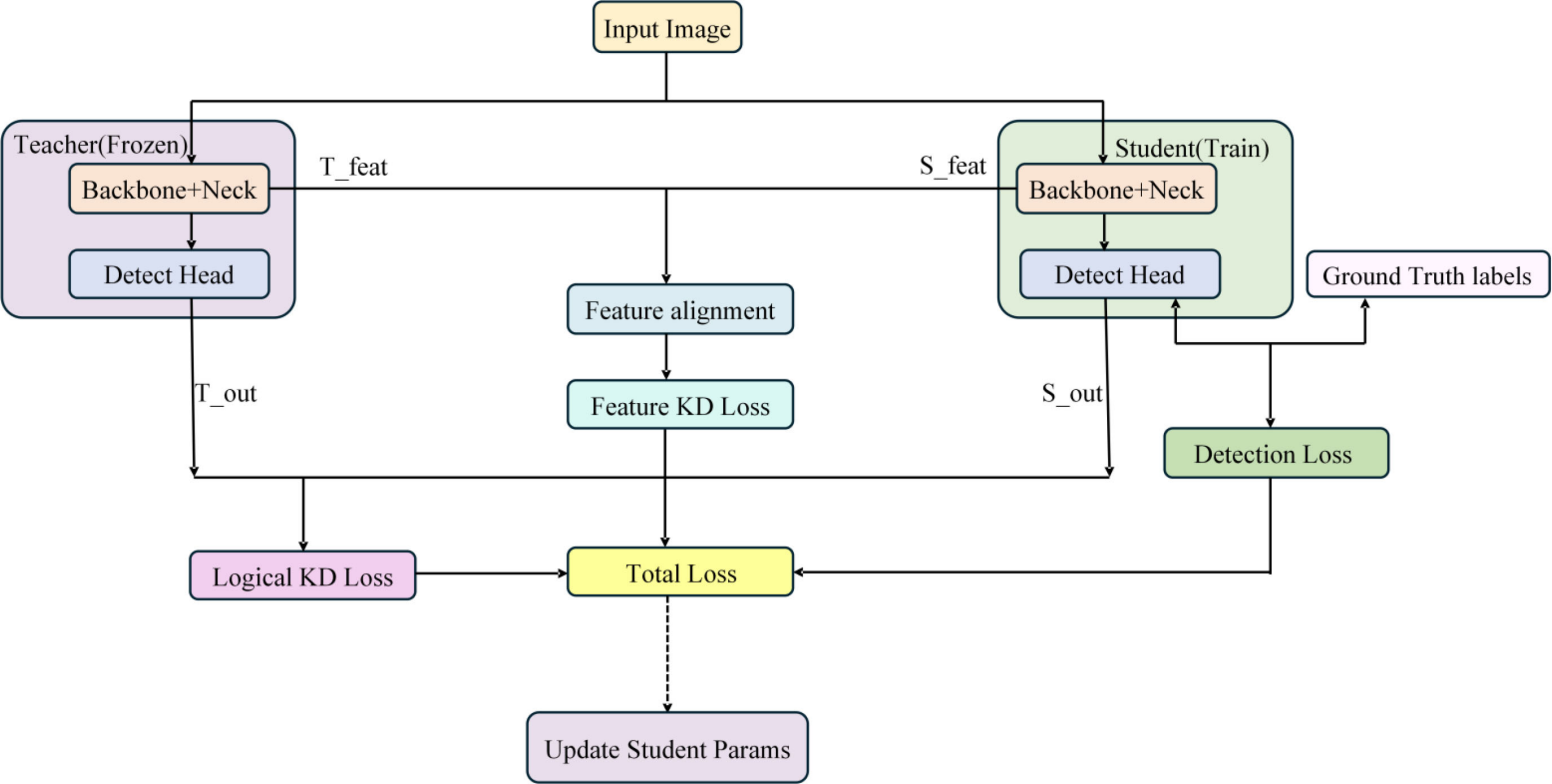
Knowledge distillation training flowchart.

**Figure 10** depicts the knowledge distillation training framework adopted in this study. Throughout training, the teacher and student models process input pictures concurrently. The instructor model’s parameters are fixed, and it only serves as a source of distillation supervision. Optimisation of the student model is guided by the combined influence of conventional detection loss and distillation loss.

The total loss function for the student model is defined as follows (Equation 15):

(15) $$\begin{array}{c} L_{total}=L_{train}+\alpha L_{CWD}+\beta L_{BCKD} \end{array}$$

In this context, $L_{train}$ refers to the standard object detection loss, while α and β denoting the weighting factors assigned to channel-level and logit distillation losses, respectively. The experimental results show that combining these two distillation strategies allows the student model to significantly improve detection performance in the blackberry ripeness detection task in complicated orchard environments while keeping low computing complexity.

### 3.2 Experimental environment and evaluation criteria

#### 3.2.1 Training environment and parameter settings

This research introduces the SDGD-YOLO network, developed atop the Ultralytics YOLO framework, for assessing the ripeness of blackberries in challenging greenhouse conditions, as detailed in **Table 2**. Experiments were carried out on an Intel Xeon Gold 6430 CPU paired with an RTX 4090 GPU (24 GB), utilising Python 3.12 in an Anaconda environment with VS Code, and employing PyTorch 2.3.0 alongside CUDA 12.1.

**Table 2:** Information on experimental hardware and software configurations.

Component	Configuration
Operating system	Ubuntu
CPU	Intel(R) Xeon(R) Gold 6430
GPU	RTX 4090(24GB)
Compiler	Anaconda + VS Code
Python version	3.12
Deep learning framework	2.3.0
CUDA Version	12.1

Model training used the hyperparameters listed in **Table 3**, with SGD optimiser (initial learning rate 0.01, momentum 0.9, weight decay 0.0005), 300 epochs, batch size 16, 4 workers, and 640×640 input resolution, while Cache and AMP were disabled. All comparison models shared these settings for fairness, and early stopping halted training if validation loss failed to improve over 50 consecutive epochs to prevent overfitting.

**Table 3:** Training hyperparameter settings.

Component	Configuration
Optimizer	SGD
Initial learning rate	0.01
Momentum	0.937
Weight decay	0.0005
Epochs	300
Workers	4
Batch	16
Input image size	640×640
Cache	False
Amp	False

#### 3.2.2 Evaluation indicators

To objectively evaluate the performance of the SDGD-YOLO network, this paper employs seven mainstream maturity object detection evaluation metrics:

Precision (P): Measures the proportion of samples that are actually in the positive class out of all samples predicted by the model to be in the positive class as shown in Equation 16.

(16) $$\begin{array}{c} P=\frac{TP}{\left(TP+FP\right)} \end{array}$$

Recall (R): This measures the proportion of samples correctly predicted as positive by the model out of all samples that are actually positive, as shown in Equation 17.

(17) $$\begin{array}{c} R=\frac{TP}{\left(TP+FN\right)} \end{array}$$

F1-Score: The harmonic mean of precision and recall; it takes both precision and recall into account and is used to balance the relationship between the two. It is expressed as follows (Equation 18):

(18) $$\begin{array}{c} F1-Score=\frac{2\times P\times R}{\left(P+R\right)} \end{array}$$

Mean Average Precision (mAP): For multi-class object detection tasks, mAP is the average of the APs for all classes. It evaluates the model’s overall detection performance across multiple classes. mAP50 refers to the mAP calculated when the IoU threshold is set to 0.5, whilst mAP50:95 refers to the average accuracy obtained by incrementally adjusting the IoU threshold from 0.5 to 0.95, reflecting the network’s robustness in object localisation and classification as shown in Equations 19–21.

(19) $$\begin{array}{c} AP=\int_{0}^{1}p(r)dr \end{array}$$

(20) $$\begin{array}{c} mAP50=\frac{1}{N}\sum_{i=1}^{\text{N}}AP_{i}(IoU_{thresh}=0.5), \end{array}$$

(21) $$\begin{array}{c} mAP50:95=\frac{1}{10}\sum_{j}(\frac{1}{N}\sum_{i=1}^{\text{N}}AP_{i}(IoU_{thresh}=j)), \end{array}$$

Parameters: The number of trainable parameters in the model. This reflects the model’s complexity. The number of parameters is a key indicator of a model’s complexity, and it has a significant impact on factors such as storage requirements, training time and computational resource consumption.

Computational efficiency (GFLOPs): The number of floating-point operations is a key metric for measuring a model’s computational load, reflecting the computational complexity of the model during inference or training.

Real-time metric: FPS refers to the number of frames per second that can be processed, and is used to measure a model’s real-time performance in practical applications as shown in Equation 22.

(22) $$\begin{array}{c} FPS=\frac{N}{T} \end{array}$$

## 4 Experimental results and analysis

### 4.1 Comparative experiment with lightweight backbone networks

**Figure 11:**
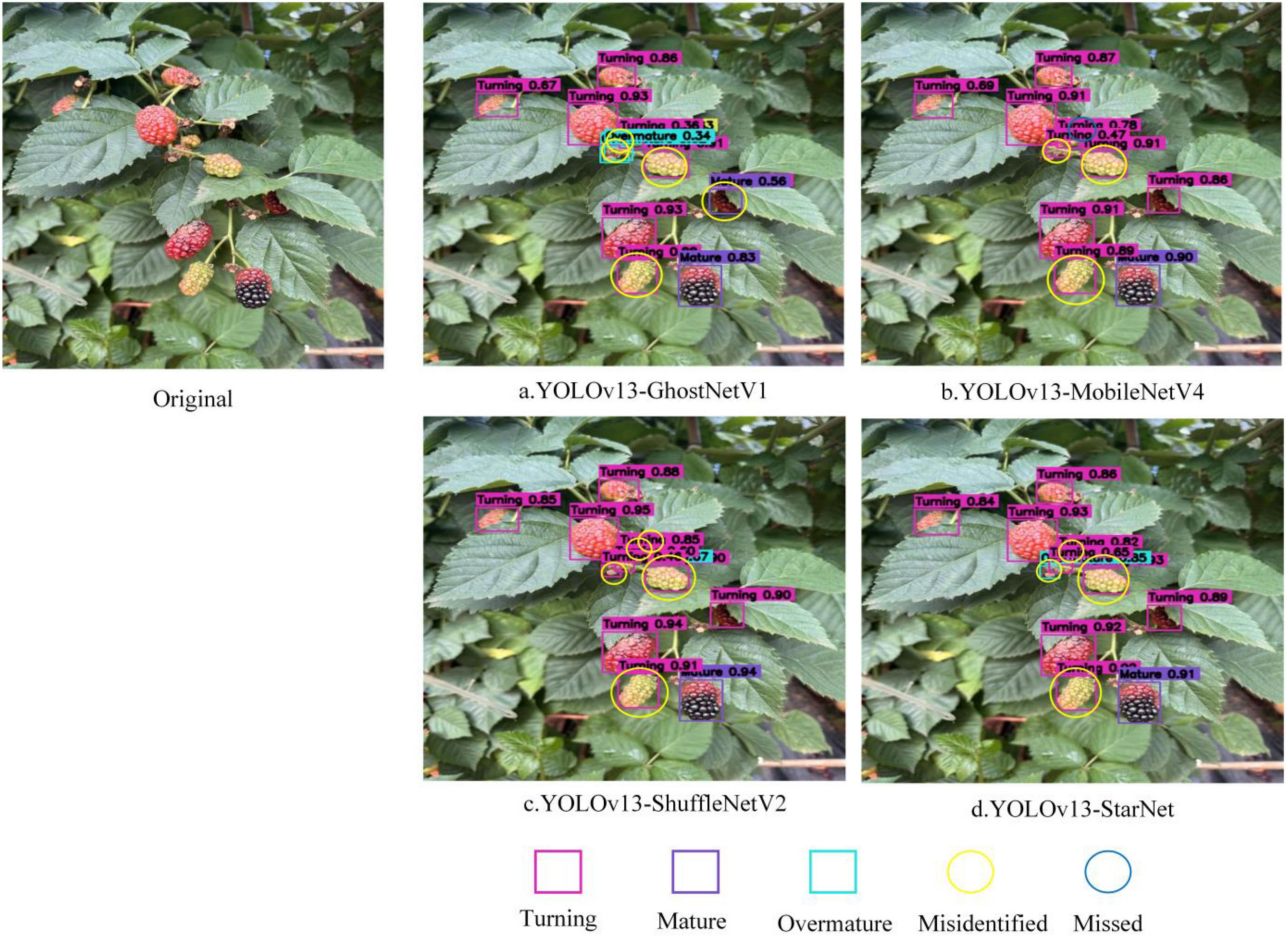
Model recognition results for different feature extraction networks.

To further validate the practical performance of different lightweight feature extraction networks in the black raspberry ripeness detection task, this study replaced the original backbone network with MobileNetV4 (Danfeng et al., 2024), ShuffleNetV2 (Ningning et al., 2018), GhostNetV1 (Kai et al., 2020), and StarNet, while keeping the rest of the YOLOv13n detection framework architecture consistent. **Table 4** shows the relevant quantitative data, whereas **Figure 11** shows the qualitative detection results. The overall metrics show a clear trade-off between detection accuracy and computational efficiency across the various lightweight networks, implying that the feature extraction architecture has a major impact on the performance of black raspberry ripeness detection.

**Table 4:** Experimental results for different lightweight backbone networks.

Model	P(%)	R(%)	mAP50(%)	mAP50:95(%)	F1-score	Parameters(M)	GFLOPs(G)	FPS
YOLOv13n	87.4	67.1	76.4	62.1	75.99	2.449	6.2	76.3
MobileNetV4	87.7	69.9	77.1	62.3	78.00	4.059	7.4	111.4
ShuffleNetV2	89.8	**72.4**	**77.7**	**63.4**	**80.00**	2.369	6.7	98.7
GhostNetV1	84.8	70.6	76.1	61.3	77.00	4.450	8.2	81.3
StarNet	**90.1**	70.7	76.2	62.3	79.10	**1.972**	**5.8**	**118.5**

The values in bold represent the best experimental results for each parameter.

As shown in **Table 4**, ShuffleNetV2 performs best on key accuracy metrics such as Recall (72.4%), mAP50 (77.7%), mAP50:95 (63.4%) and F1-Score (80.00%), demonstrating strong object detection and class discrimination capabilities, Meanwhile, its number of parameters (2.369 M) is slightly lower than that of the baseline model YOLOv13n (2.449 M), and its inference speed (98.7 FPS) shows a significant improvement over YOLOv13n (76.3 FPS), achieving a preliminary balance between accuracy and lightweight design. MobileNetV4 performs consistently across all accuracy metrics and offers a notable advantage in inference speed (111.4 FPS); however, its number of parameters (4.059 M) and GFLOPs (7.4 G) are significantly higher than those of YOLOv13n. The model’s high complexity makes it unsuitable for deployment on edge harvesting devices with limited computational power. GhostNetV1 has a higher number of parameters and computational overhead than the baseline models, and its key accuracy metrics—such as Precision, mAP and F1-Score—are generally lower. Its detection stability is insufficient, making it difficult to meet detection requirements in complex greenhouse environments. StarNet performs excellently in terms of accuracy, with a Precision (90.1%) that is the highest among all models and an F1-Score (79.10%) second only to ShuffleNetV2, whilst achieving extreme lightweight design: With only 1.972 million parameters—the lowest of all models—and 5.8 GFLOPs—also the best—it achieves the highest inference speed (118.5 FPS) of all models, striking an optimal balance between accuracy, parameter count, computational complexity and inference speed.

The visualisation results further demonstrate that there are marked differences in the detection performance of different models within complex orchard environments. As shown in **Figures 11A–C**, GhostNetV1, MobileNetV4 and ShuffleNetV2 all exhibit false positives or false negatives to varying degrees; some smaller, occluded or colour-changing fruits fail to be reliably identified, and there are also issues with confusion regarding ripeness categories. In contrast, YOLOv13-StarNet is able to detect fruit targets more comprehensively in the aforementioned scenarios, distinguishes between different stages of ripeness more clearly, and significantly reduces the number of false positives and false negatives.

Combining the quantitative results in **Table 4** with the qualitative analysis in **Figure 11**, we can infer that StarNet greatly decreases the number of model parameters and computing complexity while retaining detection accuracy. With only 1.972 million parameters, 5.8 GFLOPs, and an inference speed of 118.5 FPS, it strikes a solid balance across several measures. As a result, StarNet is more useful in the application situation of detecting black raspberry ripeness, which is marked by’small targets, high occlusion, and high inter-class similarity’. As a result, the backbone network for SDGD-YOLO in this study is StarNet.

### 4.2 Comparative testing of Soft-NMS

To investigate the differences between Soft-NMS and standard NMS in the black raspberry ripeness detection task, this experiment used a variety of Soft-NMS methodologies for comparative testing, while retaining consistent benchmark model settings. These comprised Soft-NMS approaches based on IoU, CIoU, SIoU, EIoU, DIoU, and GIoU, with detection results presented in **Table 5**.

**Table 5:** Comparative experiments of Soft-NMS versus traditional NMS with different loss functions.

NMS strategy	mAP50 (%)	mAP50:95 (%)
NMS	76.4	62.1
Soft-NMS-IOU	80.0	68.1
Soft-NMS-CIOU	80.1	68.3
Soft-NMS-SIOU	79.5	67.6
Soft-NMS-EIOU	79.5	67.7
Soft-NMS-DIOU	79.7	68.3
Soft-NMS-GIOU	**80.4**	**68.5**

The values in bold represent the best experimental results for each parameter.

The data in the table show that traditional NMS performs much worse than other Soft-NMS approaches on both the mAP50 and mAP50:95 measures. This suggests that, in real-world orchard scenarios with dense fruit clusters and extreme object overlap, actively suppressing overlapping targets might easily lead to the incorrect removal of legitimate detection boxes, resulting in detection omissions. In contrast, Soft-NMS improves overall detection performance by continuously attenuating the confidence scores of candidate bounding boxes.

Among the several Soft-NMS variations, Soft-NMS-GIoU outperformed the others in terms of overall performance, with a mAP50 of 80.4% and a mAP50:95 of 68.5%. This suggests that in the blackberry ripeness detection scenario, where high occlusion and small targets coexist, incorporating GIoU’s boundary constraint information helps to more accurately characterise the spatial relationship between predicted and ground-truth bounding boxes, improving the rationality of bounding box ranking and retention.

Soft-NMS-GIoU improves the capacity to differentiate highly overlapping fruit targets while retaining algorithmic stability, thereby addressing the issue of missed detections in crowded scenes that classic NMS suffers from. As a result, in the following studies, this study used Soft-NMS-GIoU as the last post-processing technique to enhance the overall accuracy and resilience of blackberry ripeness detection.

### 4.3 Ablation experiments

To confirm the effectiveness of each enhanced module in the black raspberry ripeness detection job, this study performed ablation tests on three important enhancements: DySample, Soft-NMS-GIoU, and the StarNet backbone network. While leaving all other variables the same, the major components were removed one at a time from the model to see how performance and complexity metrics changed; the results of these tests are reported in **Table 6**.

**Table 6:** Melting experiment results.

YOLOv13n	DySample	Soft-NMS-GIOU	StarNet	P(%)	R(%)	mAP50(%)	mAP50:95(%)	F1-score	Parameters(M)	GFLOPs(G)	FPS
√	**×**	**×**	**×**	87.4	67.1	76.4	62.1	75.99	2.449	6.2	76.3
√	√	**×**	**×**	87.0	**71.3**	76.8	62.5	78.40	2.465	6.2	**71.8**
√	**×**	√	**×**	87.3	70.1	80.4	68.5	77.84	2.489	6.2	78.4
√	**×**	**×**	√	90.1	70.7	76.2	62.3	79.10	1.972	5.8	118.5
√	√	√	**×**	89.1	69.7	80.8	69.4	78.35	2.465	6.2	72.4
√	√	**×**	√	90.3	70.6	76.4	62.8	79.20	1.982	5.8	106.5
√	**×**	√	√	90.2	69.7	80.7	69.2	78.03	**1.972**	5.8	117.3
√	√	√	√	**91.3**	70.7	**82.0**	**70.8**	**79.87**	1.988	**5.8**	99.6

The values in bold represent the best experimental results for each parameter.

As indicated in the table, while utilising the original YOLOv13n as the baseline model, its accuracy on the test set was 87.4%, recall was 67.1%, and mAP50 and mAP50:95 were 76.4% and 62.1%, respectively; however, the detection performance still showed some false negatives in complex orchard scenarios. Following the implementation of DySample, the model’s recall rate went from 67.1% to 71.3%, while the F1-score increased to 78.40, demonstrating that the dynamic sampling method improved the model’s capacity to detect occluded and edge-of-frame fruits to some extent. However, the gain in mAP measurements was modest, and detection accuracy actually declined. When Soft-NMS-GIoU was included alone, the model showed a significant improvement in the mAP metric, with mAP50 rising to 80.4% and mAP50:95 rising to 68.5%, reflecting gains of 4.0% and 6.4% over the baseline model. This suggests that in cases where blackberry fruits are densely grouped and have severe growth overlap, the GIoU-based Soft-NMS can keep valid detection boxes in highly overlapping regions, considerably reducing false negatives caused by standard NMS. However, its impact on model complexity and inference performance is negligible, with the number of parameters and GFLOPs remaining relatively constant. After replacing the backbone network with StarNet, the model’s benefits in terms of lightweight design and real-time performance became clear. The number of parameters was lowered dramatically from 2.449 million to 1.972 million, GFLOPs fell to 5.8, and FPS climbed to 118.5, resulting in a significant improvement in inference performance. In terms of detection performance, accuracy increased to 90.1%, and the F1-score reached 79.10, indicating that StarNet effectively reduces the model’s computational burden while maintaining high detection accuracy, making it ideal for deployment on edge devices with limited computing power.

When DySample, Soft-NMS-GIoU and StarNet are introduced simultaneously, the model achieves an optimal balance between accuracy and efficiency. Accuracy increases to 91.3%, recall reaches 70.7%, and mAP50 and mAP50:95 rise to 82.0% and 70.8% respectively—all of which are the highest values recorded across all comparative experiments. Meanwhile, the number of parameters is kept at 1.988 M, GFLOPs remain at 5.8, and FPS stands at 99.6. Ablation results indicate that FPS has decreased significantly compared to using StarNet alone (118.5), primarily due to the additional feature computation and interpolation overhead introduced by the DySample dynamic upsampling module; whereas Soft-NMS-GIoU had virtually no impact on inference speed. Despite this slight reduction in speed, 99.6 FPS remains significantly higher than the baseline model’s 76.3 FPS, meeting the real-time requirements for practical detection. The experimental results demonstrate that the improvements to the YOLOv13n model proposed in this study are synergistically effective.

**Figure 12:**
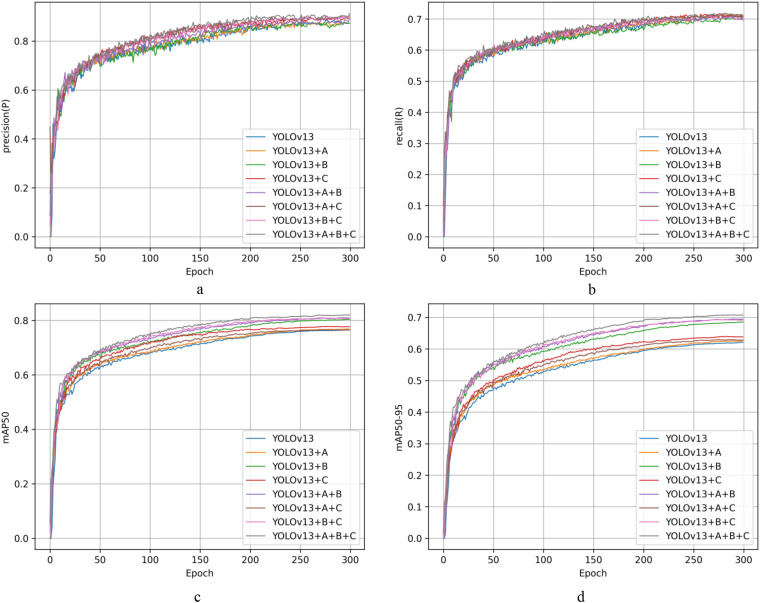
Performance metric variation curves for different module combinations across distinct training periods. Capital letter A denotes DySample, capital letter B denotes Soft-NMS-GIoU, and capital letter C denotes StarNet. **(a)** denotes the precision curve, **(b)** denotes the recall curve, **(c)** denotes the mAP50 curve, **(d)** denotes the mAP50:95 curve.

**Figure 12A** shows the accuracy trends of different module combinations at various training stages; **Figure 12B** shows the recall curves of different module combinations at different training stages; and **Figures 12C, D** show the dynamic changes in mAP50 and mAP50:95 for different module combinations during training. Based on these four basic measures, it is clear that the comprehensive upgraded model beats competing module combinations in all areas. This result verifies the generalised model’s improved comprehensive performance in the blackberry ripeness detection task in terms of dynamic training. The extensive integration of its lightweight backbone network with the post-processing mechanism allows it to perform more robustly and adaptively in blackberry ripeness detection jobs in complex scenarios.

### 4.4 The impact of knowledge distillation on model performance

This study also used a knowledge distillation strategy to train the model by adding the improved model’s properties, which enhanced its performance even further. By transferring knowledge from the instructor model to the student model, the student model may better acquire and apply the knowledge provided through distillation, resulting in improved performance. The student model was YOLOv13n_SDG, which was selected through ablation experiments. To choose the most appropriate instructor model, comparisons were done with YOLOv13l_SDG, YOLOv13x_SDG, and YOLOv13s_SDG from the YOLOv13 series. **Table 7** shows the performance of the YOLOv13_SDG series of models.

**Table 7:** Performance comparison of YOLOv13_SDG series models.

Model	P (%)	R (%)	mAP50 (%)	mAP50:95 (%)	F1-score	Parameters (M)	GFLOPs (G)
YOLOv13n_SDG	91.3	70.7	82.0	70.8	79.87	1.988	5.8
YOLOv13l_SDG	**94.4**	72.5	83.7	76.9	82.1	17.537	50.5
YOLOv13x_SDG	93.1	**73.9**	**83.7**	**78.3**	**82.4**	40.946	**113.2**
YOLOv13s_SDG	91.7	71.8	82.2	73.0	80.54	**58.627**	14.7

The values in bold represent the best experimental results for each parameter.

As model size rises, both YOLOv13l-SDG and YOLOv13x-SDG show considerable improvement in detection performance. In instance, YOLOv13l-SDG has a Precision score of 94.4% and a mAP50 of 83.7%, indicating great overall detection accuracy. However, the recall increase is limited, and the model’s capacity is still insufficient to capture the different properties of objects in complicated situations. In comparison, YOLOv13x-SDG has the best overall performance across a variety of critical criteria. Its recall is 73.9%, and its F1-score is 82.4%. It also scores 83.7% and 78.3% for the mAP50 and mAP50:95 measures, respectively, ranking highest among all models. This suggests that YOLOv13x-SDG has improved feature representation and object classification skills, allowing it to gather more detailed information about objects in complicated scenarios. Although YOLOv13x-SDG has significantly more parameters and higher computational complexity than other models, in knowledge distillation tasks, the performance ceiling of the teacher model has a decisive influence on the distillation results. Stronger feature representation capabilities help to transfer richer and more discriminative knowledge to the student model, thereby compensating for the performance loss resulting from the lightweight design.

Based on detection accuracy, recall performance, and feature representation capabilities, YOLOv13x-SDG was chosen as the instructor model in this study’s knowledge distillation procedure.

To efficiently transfer knowledge from the teacher model YOLOv13x-SDG to the student model YOLOv13n_SDG, we chose numerous levels of the model for distillation, with a focus on the P3, P4, and P5 feature layers, which operate at varying resolutions. The P3 layer represents high-resolution, detailed characteristics, allowing the student model to better capture the minute details of small objects while also displaying increased robustness, especially in complicated backgrounds. The P4 layer functions as a mid-scale semantic transition layer, effectively combining information from various scales to improve the model’s detection skills for medium-sized items. The P5 layer represents higher-level global semantic information, which aids the student model’s acquisition of global contextual aspects as well as its capacity to localise and identify huge objects. This method overcomes the information bias associated with single-layer distillation, while also improving the student model’s capacity to represent fine-scale details, model scale, and structure under complicated occlusions, and make global semantic judgements on maturity categories. By performing knowledge distillation across these layers, the student model improves its ability to learn the teacher model’s feature representations at various scales, as well as its accuracy in fine-grained object recognition and complex background settings.

The model created using knowledge distillation is called YOLOv13n_SDG+KD, and the distillation results are reported in **Table 8**. The experimental results show that, whilst maintaining the same number of parameters and computational complexity, YOLOv13n_SDG+KD achieves a recall rate 1.1 percentage points higher and an F1 score 0.81 points higher than YOLOv13n_SDG, thereby effectively reducing the number of missed black raspberry fruits. Although the average precision reduces significantly at high IoU thresholds, this improvement better fulfils the actual application requirements for detecting black raspberry ripeness.

**Table 8:** Knowledge distillation results.

Model	P (%)	R (%)	mAP50(%)	mAP50:95(%)	F1-score	Parameters(M)	GFLOPs(G)
YOLOv13n	87.4	67.1	76.4	62.1	75.99	2.449	6.2
YOLOv13n_SDG	**91.3**	70.7	**82.0**	**70.8**	79.87	1.988	5.8
YOLOv13n_SDG+KD	91.0	**71.8**	81.7	68.7	**80.68**	**1.988**	**5.8**

The values in bold represent the best experimental results for each parameter.

**Figure 13:**
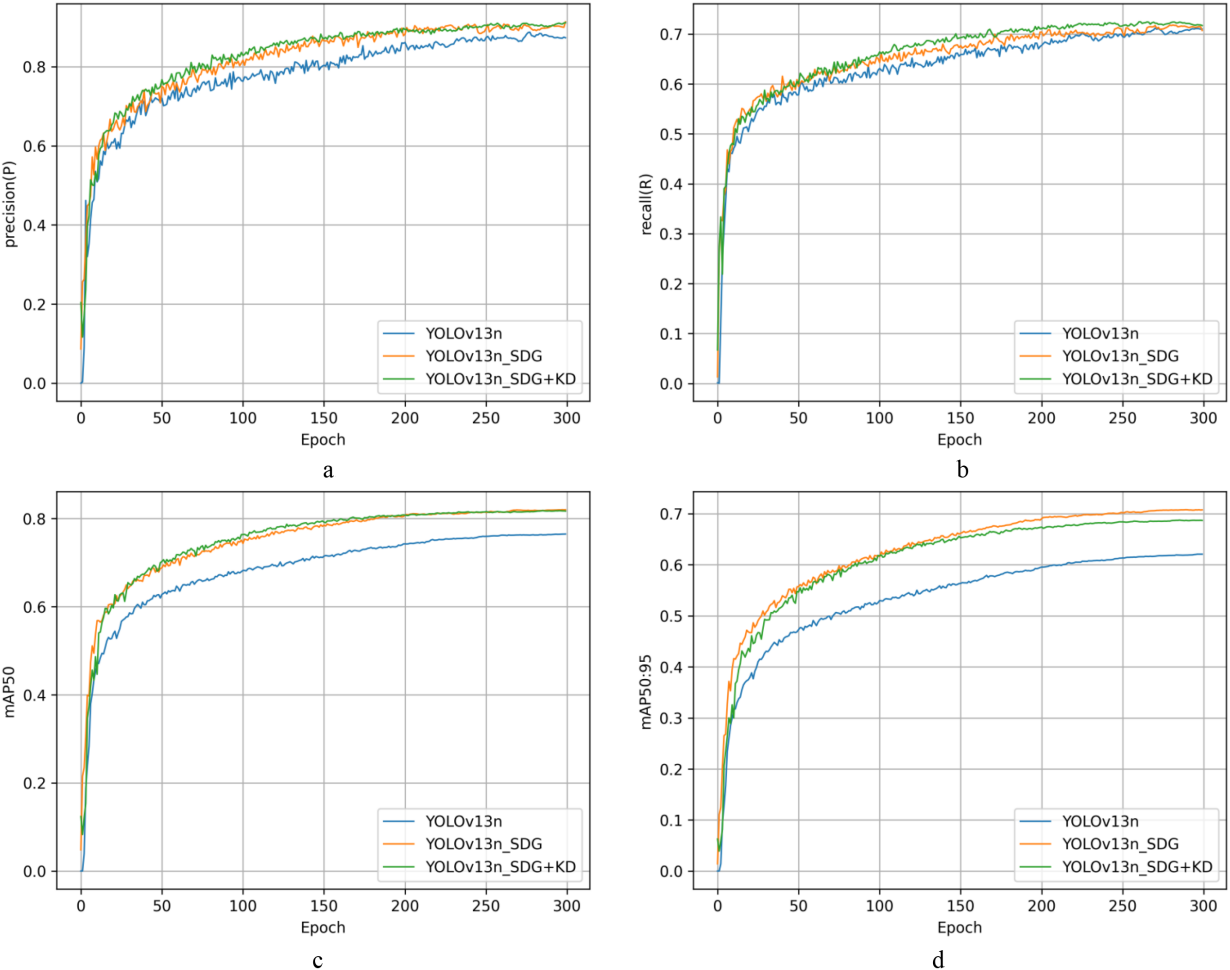
Performance metric variation curves across different training periods before and after model refinement.

**Figure 13** shows the accuracy curves before and after model improvement. As shown by the accuracy curves, the accuracy of all three models increased fast during the early phases of training before stabilising in the middle and later stages, demonstrating that the network was able to learn effective discriminative features very quickly. In particular, both YOLOv13n-SDG and YOLOv13n-SDG+KD have much greater overall accuracy levels than the original YOLOv13n, and the enhanced models maintain higher accuracy throughout the majority of the training stages, showing their superior capacity to suppress false positives. In the recall curves, all three models show a pattern of quick initial improvement followed by continuous convergence. Compared to the baseline model, YOLOv13n-SDG+KD, which includes structural enhancements and knowledge distillation, maintains a high recall rate throughout the training process and achieves the highest stable value in the later stages. This shows that the enhanced model can detect more genuine objects in difficult circumstances, effectively lowering false negatives. The mAP50 curve shows that the upgraded model had a noticeable advantage in the early phases of training and converged faster than the baseline model. After around 200 epochs, all models’ performance stabilised, although the mAP50 of YOLOv13n-SDG and YOLOv13n-SDG+KD remained consistently higher than that of YOLOv13n. Notably, the model that included knowledge distillation had a smoother overall curve and a modest improvement in final accuracy, suggesting the positive influence of the distillation technique on detection performance. The results of the complete performance curves reveal that the YOLOv13n-SDG+KD model surpasses the comparison models in terms of convergence speed, stability, and final detection performance, demonstrating the proposed method’s efficacy and superiority.

### 4.5 Comparative experiments on different network models

To validate the SDGD-YOLO model’s overall performance in the black raspberry ripeness detection task, comparative experiments were carried out under identical experimental conditions (including the same dataset, training strategy, and parameter settings), pitting the SDGD-YOLO model against a variety of mainstream object detection methods. These included the single-stage end-to-end real-time object detection model RT-DETR, as well as lightweight single-stage YOLO models including YOLOv3-tiny, YOLOv5n, YOLOv6n, YOLOv8n, YOLOv9t, YOLOv10n, YOLOv11n, and YOLOv13n. **Table 9** shows the experimental outcomes.

**Table 9:** Comparison of results from different model tests.

Model	P (%)	R (%)	mAP50(%)	mAP50:95(%)	F1-Score	Parameters(M)	GFLOPs(G)	FPS
RT-DETR	88.5	69.0	72.7	58.2	77.54	32.000	103.4	36.4
YOLOv3-tiny	88.8	**72.6**	80.0	69.2	79.89	12.130	18.9	**638.0**
YOLOv5n	85.8	68.6	78.9	67.4	76.24	2.504	7.1	303.8
YOLOv6n	86.4	68.6	78.9	67.8	76.48	4.234	11.8	309.0
YOLOv8n	89.6	72.0	81.2	70.2	79.86	3.006	8.1	311.4
YOLOv9t	88.5	70.4	80.6	**69.5**	78.42	**1.972**	7.6	123.5
YOLOv10n	88.1	70.0	78.7	63.1	78.01	2.696	8.2	192.7
YOLOv11n	86.5	70.4	78.8	62.9	77.62	2.583	6.2	225.5
YOLOv13n	87.4	67.1	76.4	62.1	75.99	2.449	6.2	76.3
Our	**91.0**	71.8	**81.7**	68.7	**80.68**	1.988	**5.8**	99.6

The values in bold represent the best experimental results for each parameter.

Several lightweight object identification models were rigorously evaluated based on measures such as accuracy, recall, F1 score, number of parameters, and computational economy. In high-precision circumstances, the SDGD-YOLO model performed admirably, with an accuracy of 91.0%, a mAP50 of 81.7%, and a mAP50:95 of 68.7%, proving its exceptional detection skills. The F1 score was 80.68, indicating a solid mix of high precision and recall. Among all the models, the SDGD-YOLO model had the fewest parameters, the lowest GFLOPs, and a lower FPS than the others. YOLOv3-tiny attained the highest FPS, but it had a large number of parameters and a higher level of model complexity, and its detection accuracy was much worse than the model reported in this study. Based on the results of the preceding trials, the model proposed in this paper has excellent accuracy, low computational complexity, and a low false negative rate. While maintaining high detection accuracy, it achieves 99.6 FPS, balancing real-time performance and accuracy. This makes it ideal for practical edge computing applications in agriculture, such as detecting ripe blackberries.

**Figure 14:**
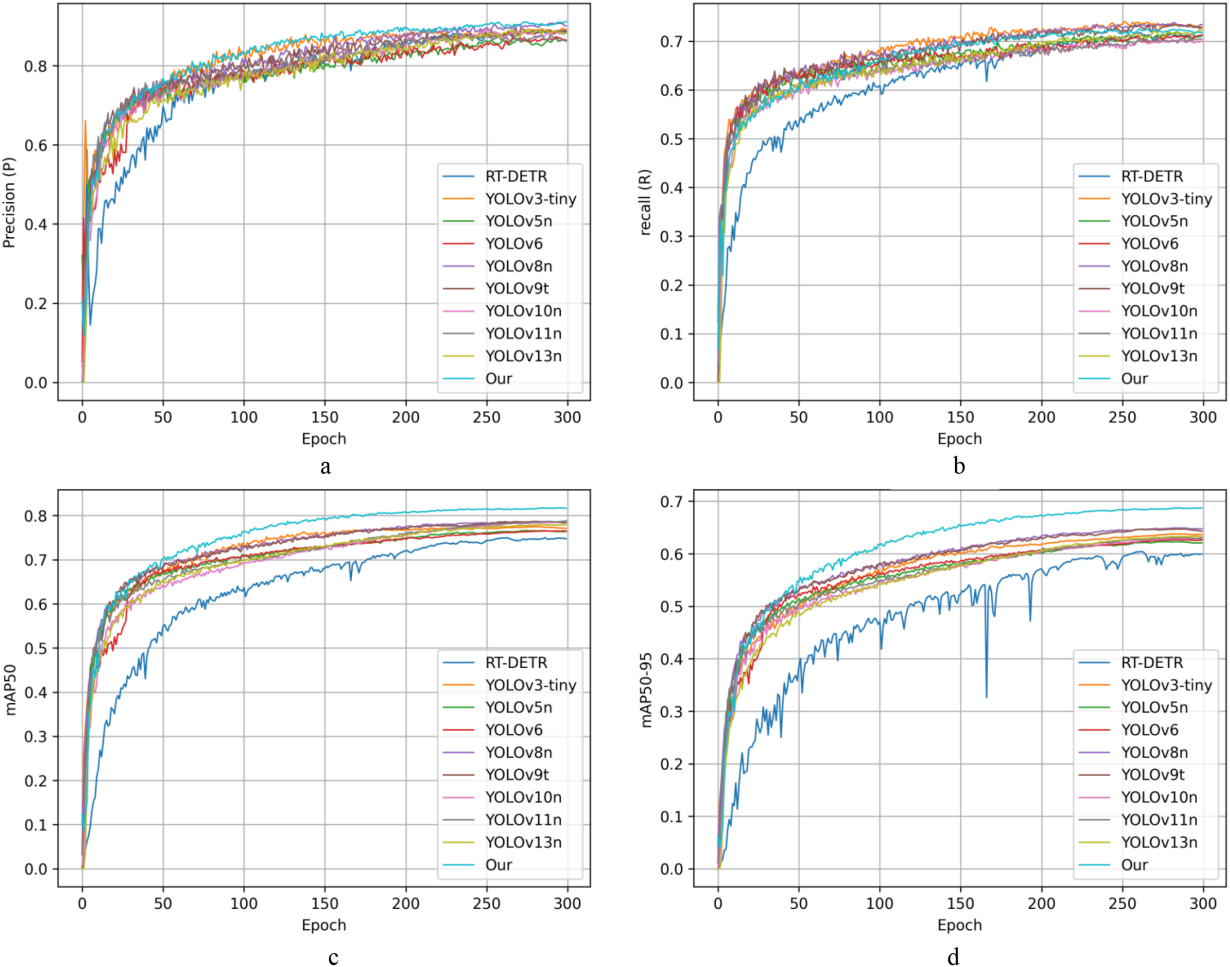
Performance metric variation curves for different models across distinct training periods.

**Figure 14** depicts the changes in performance measures for each model on the same dataset during the training process, as well as the end training outcomes. **Figure 14A** depicts the accuracy curves for various models in the training phase. In the early phases of training, the model suggested in this study displayed a consistent improvement in accuracy when compared to other models, and it continued to develop steadily in the later stages, eventually obtaining the best results, greatly exceeding all others. **Figure 14B** depicts the recall curves of multiple models at different phases of training. The recall performance of the model presented in this study increases in the early stages in the same way that previous models do, but, in the latter stages, its recall continues to rise, exhibiting significant adaptability and stability. In contrast, other models frequently plateau or drop during this stage; the SDGD-YOLO model efficiently avoids false negatives caused by feature confusion, resulting in a higher recall rate. **Figures 14C, D** depict mAP50 and mAP50:95 curves for several models as training advances, respectively. The SDGD-YOLO model converges quickly in the early phases of training and continues to optimise in the middle and later stages, constantly maintaining excellent performance that outperforms other models. Under varying accuracy criteria, the SDGD-YOLO model performs well, retaining strong detection skills in complicated backdrops while meeting high accuracy standards in both localisation and classification tasks, resulting in the best overall detection performance.

This study employs gradient-based activation maps (Grad-CAM) to systematically evaluate the visual interpretability of an improved blackberry ripeness model. Grad-CAM obtains importance weights for each channel by globally averaging the gradients of the feature maps corresponding to the target class, and then performs a weighted fusion of these weights with the original feature maps to generate a class activation heatmap, thereby visualising the key regions of interest to the model during the prediction process (Rathi and Nirgude, 2023). As illustrated in the picture, the heatmap’s highly activated regions (red areas) correspond to the model’s discriminative confidence in the target’s essential features, suggesting that the model can effectively focus on discriminatively significant regions in complicated contexts.

**Figure 15:**
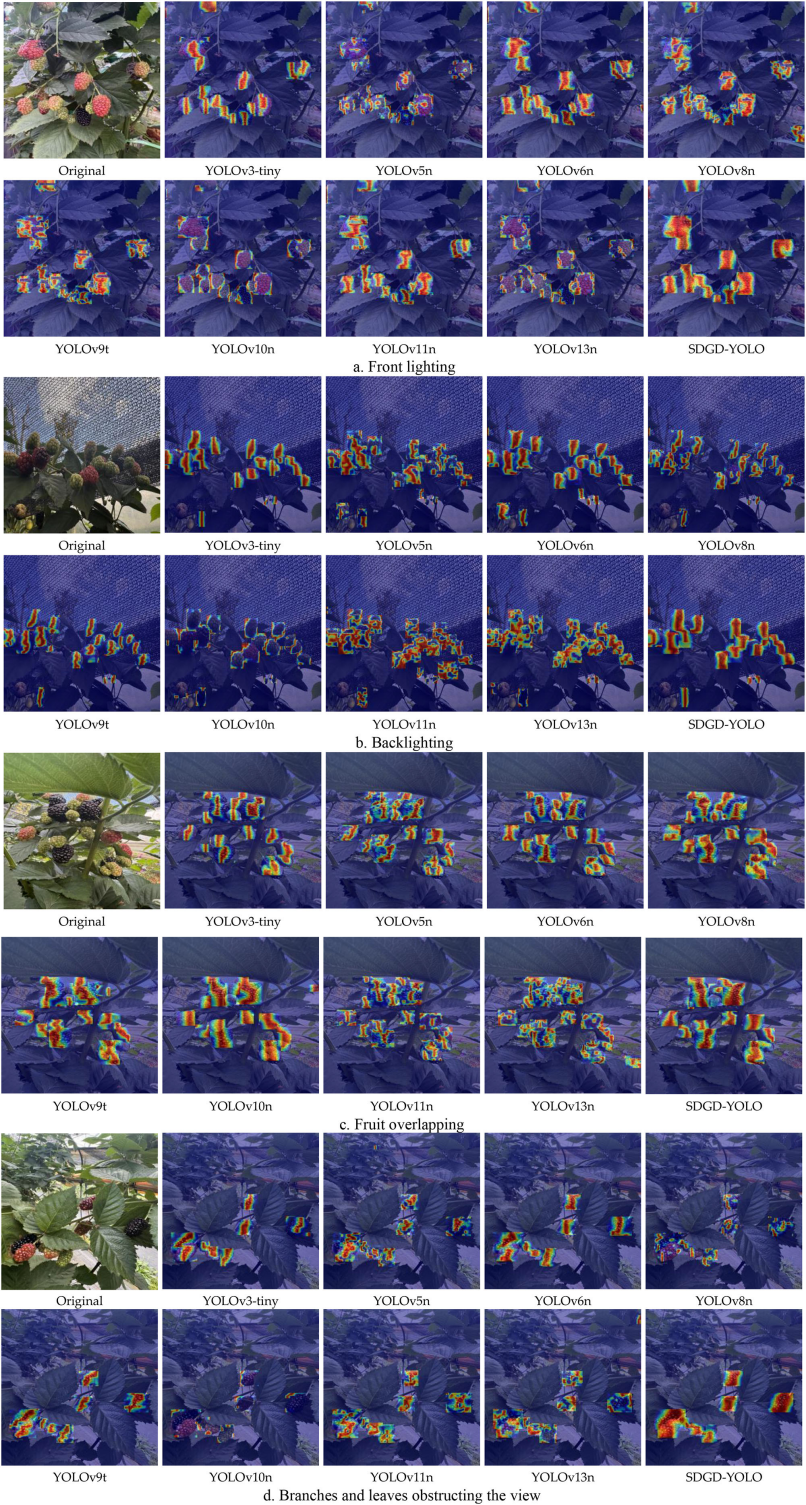
Visualisation heatmaps for model inference experiments.

The Grad-CAM heatmap findings are displayed in **Figure 15**. **Figure 15A** displays a front-lit situation with the fruit’s surface texture clearly visible and obvious boundary features. The model’s high-response regions are predominantly located in the fruit’s main body, demonstrating that the model can completely utilise colour and texture information for ripeness classification, with relatively reliable detection findings; **Figure 15B** displays a backlighting scenario. Due to the bright illumination, certain sections of the fruit are overexposed or in shadow, diminishing the contrast between the target and the background. The model’s attention distribution is relatively dispersed, which complicates feature extraction and introduces the possibility of misclassification. **Figure 15C** depicts a scenario in which several fruits conceal one another, and their boundaries overlap greatly, reducing the distinguishing contour features of individual fruits. The model must rely on local prominent cues for classification, which can easily result in missed or incorrect detections; **Figure 15D** displays a scenario in which the fruit is concealed by branches and leaves. These branches and leaves partially cover the fruit spatially, interfering with the expression of its morphological and colour traits, making it more difficult for the model to distinguish the target from the backdrop.

This study model has substantial advantages for suppressing complicated background interference and recognising objects in dense situations. Compared to standard YOLO models, the heatmap feature distribution is more focused. **Figure 15A** shows that in the Grad-CAM heatmap of a backlit scene, the SDGD-YOLO model greatly minimizes its response to background noise; the hot regions precisely cover the margins of the raspberries, with a uniform and continuous intensity distribution. In contrast, YOLOv3-tiny shows scattered hotspots with significant background response; YOLOv5n shows fragmented hotspots; YOLOv8n has hotspots that are too large and include background noise; and YOLOv10n has hotspots that are overly concentrated, resulting in some small objects not being activated. None of these models can match the feature-focused performance of SGDG-YOLO.

In backlit scenarios (**Figure 15B**), when high illumination diminishes the contrast between the fruit and the backdrop, models like YOLOv3-tiny, YOLOv5n, and YOLOv6n show scattered or fragmented hotspots, resulting in significant background misclassification and unstable feature extraction. The SDGD-YOLO model’s Grad-CAM heatmap shows red patches that entirely cover all visible fruits, with clear and sharp borders and great background suppression. Even in shaded locations, the model can accurately localise the fruits, demonstrating that the SDGD-YOLO model is more adaptable to lighting conditions and has superior feature stability.

In the challenge featuring overlapping fruit (**Figure 15C**), the raspberries overlap and impede one another, with some overlapping by more than 50% and their boundaries becoming obscured. The YOLOv3-tiny model’s heatmaps show extensive merging, making it impossible to distinguish individual berries; the YOLOv5n model’s heatmaps suffer from severe clustering, resulting in the complete loss of boundaries; the YOLOv8n model’s heatmaps exhibit both fragmentation and clustering; and the YOLOv11n model’s heatmaps contain obvious gaps, leading to significant missed detections. The SDGD-YOLO model’s heatmap accurately separates each individual fruit within the red regions, with clear and distinguishable boundaries; even in overlapping areas, independent activation is maintained, effectively resolving the issue of blurred boundaries caused by mutual occlusion between fruits in dense scenes.

In situations with foliage occlusion (**Figure 15D**), the raspberries are heavily concealed by branches and leaves, leaving just sections visible, resulting in extremely fragmentary characteristics. YOLOv3-tiny’s hotspots only cover unoccluded areas; YOLOv5n and YOLOv6n do not activate for occluded fruits; YOLOv8n exhibits positioning shifts and shape distortion; and YOLOv10n exhibits almost no response in occluded areas. The SDGD-YOLO model’s heatmap fully covers the occluded fruit, using contextual inference to fill in the occluded areas. In the case of an object heavily obscured by leaves in the bottom-right corner of the image, it detects both objects simultaneously, whereas other models either focus on only one object or are completely unable to generate a heatmap for the obscured object. This demonstrates that the SDGD-YOLO model achieves a high degree of accuracy in feature recognition and object localisation.

**Figure 16:**
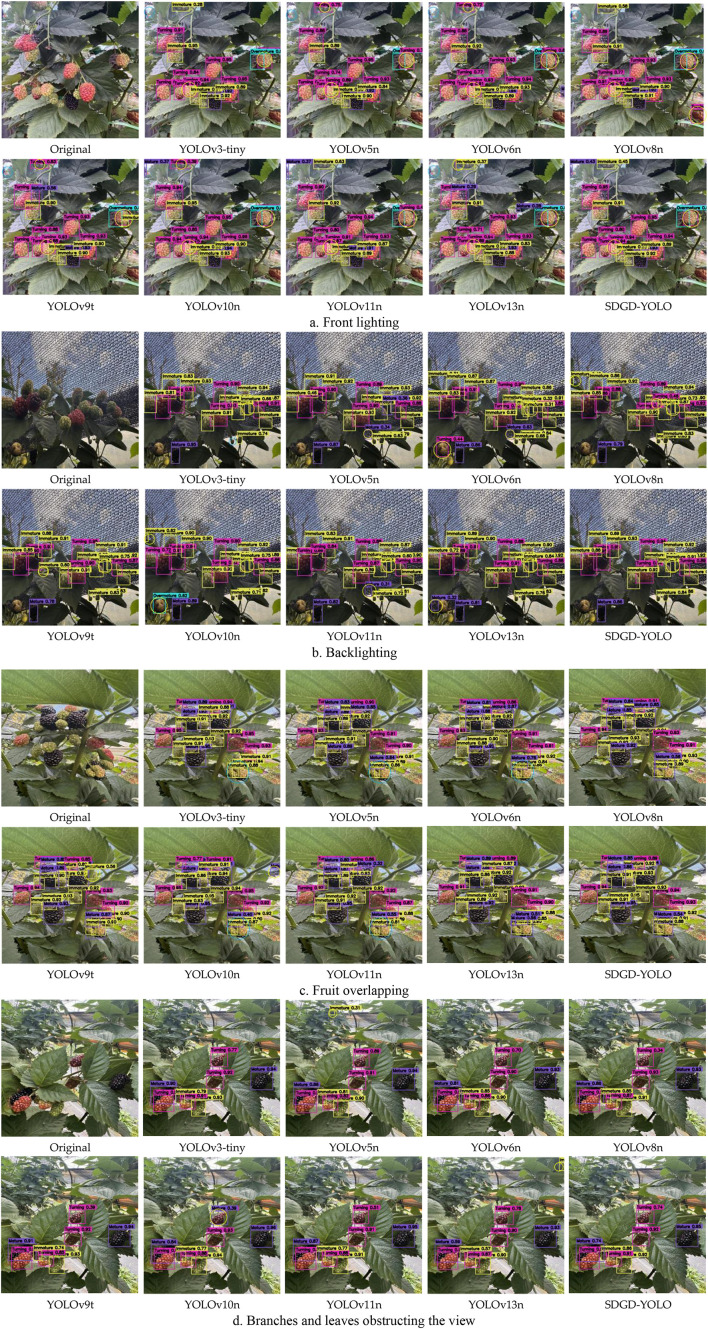
Visualisation results of inference experiments for each model.

To assess the SDGD-YOLO model’s detection performance, we visually compared the detection results of each model across different scenarios, resulting in an intuitive representation of each model’s detection boundaries, confidence distributions, and false negative/false positive rate. **Figure 16** shows the results. **Figure 16A** depicts a backlit image with an occluded section of black raspberries in the top-left corner, fruit overlap in the centre, and a complicated background in the bottom-right. Only the YOLOv10n, YOLOv11n, and SDGD-YOLO models could detect the partially veiled blackberries in the top-left corner; however, YOLOv10n and YOLOv11n misidentified the unripe blackberries as blackberries in the colour-changing stage. Among the models that did not misclassify, SDGD-YOLO had the best confidence. Under **Figure 16B**, YOLOv5n, YOLOv6n, YOLOv8n, YOLOv10n, YOLOv11n, and YOLOv13n all misidentified pears under protective netting or complicated backdrops as black raspberries, but the confidence distributions of the other models were unequal. In contrast, the SDGD-YOLO model exhibited stable detection performance under these complicated illumination conditions, with the lowest false negative rate and more thorough coverage of densely clustered fruit. Furthermore, it gave reasonable confidence annotations for raspberries at various stages of ripeness, demonstrating the model’s greater responsiveness to lighting conditions and feature robustness. **Figure 16C** shows black raspberries closely crowded, with significant overlap and obstruction; in some cases, the overlap surpasses 50%. Furthermore, the presence of greenery covering the borders results in fuzzy boundaries, putting tremendous strain on the model’s instance segmentation and individual recognition abilities. YOLOv3-tiny, YOLOv5n, YOLOv6n, YOLOv10n, and YOLOv11n all missed the black raspberry on the right, which was obscured, yielding a false negative. YOLOv8n, YOLOv9t, and YOLOv13n identified the black raspberry on the right, which was partially obscured twice, resulting in overlapping bounding boxes. Furthermore, most models produced false positives and poor confidence scores. Overall, the SDGD-YOLO model outperformed the other models in this dense scenario due to optimised feature extraction and transfer learning; it maintained independent detection even in overlapping regions and had a substantially lower false negative rate. **Figure 16D** shows the raspberry fruit shrouded by leaves, with only a piece visible, resulting in highly inadequate feature information. YOLOv5n incorrectly identified the branches and leaves as black raspberries, while YOLOv10n misidentified the upper central area—which was in the colour-changing stage—as a ripe black raspberry, YOLOv13n identifies the background as fruit. Among the remaining models that did not misclassify, Among the remaining models that did not misclassify, the SDGD-YOLO model had the highest confidence scores, which were likewise uniformly distributed.

**Figure 17:**
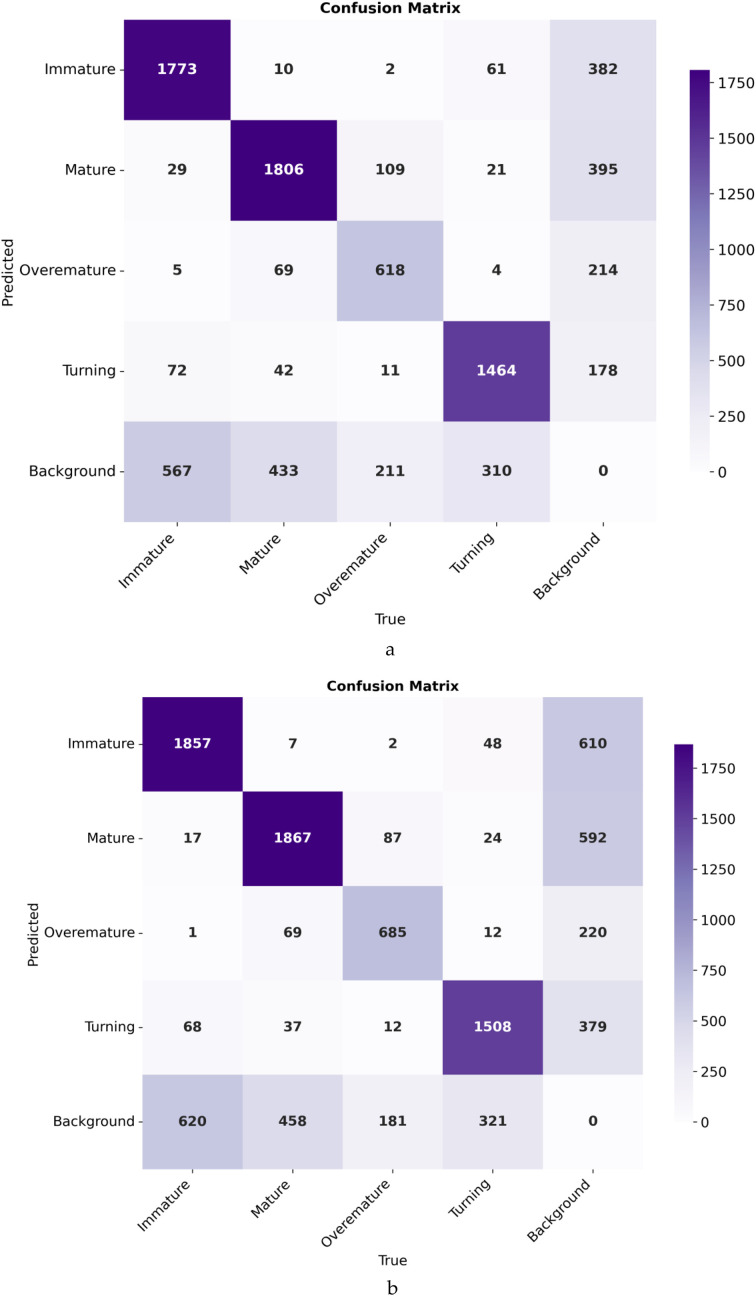
Comparison of the confusion matrix for YOLOv13n with the SDGD-YOLO model.

Based on the confusion matrix shown in **Figure 17**, the SDGD-YOLO model demonstrates a significant improvement in performance compared to the YOLOv13n model in the black raspberry ripeness detection task. The confusion matrix allows for an intuitive assessment of the model’s classification performance across different ripeness categories, with robustness in multi-class fruit detection—particularly in complex greenhouse environments—being comprehensively enhanced. In the detection of unripe blackberries, the SDGD-YOLO model correctly identified 1,857 unripe samples, significantly higher than the 1,773 identified by the YOLOv13n model. Furthermore, it misclassified fewer genuine unripe fruits into other ripeness categories, indicating that SDGD-YOLO performs more precise feature extraction for unripe fruits, thereby effectively enhancing its detection capability in complex environments. In the detection of ripe blackberries, the SDGD-YOLO model correctly identified 1,867 ripe samples, markedly higher than the 1,806 identified by the YOLOv13n model. Furthermore, it misclassified fewer ripe fruits into other categories such as the colour-changing stage or overripe stage, indicating that SDGD-YOLO possesses a distinct advantage in distinguishing ripe fruit categories. It is better equipped to handle background interference and fruit occlusion issues, thereby enhancing overall detection accuracy. Regarding black raspberries in the colour-changing stage—a key category characterised by ambiguous features and prone to confusion— the SDGD-YOLO model correctly identified 1,508 samples, significantly outperforming the 1,464 samples correctly identified by the YOLOv13n model. At the same time, it substantially reduced misclassifications of fruits in the colour-changing stage with other ripeness categories and the background, effectively resolving the high false positive rate issues of YOLOv13n in scenarios involving overlapping fruits and small targets, demonstrating stronger target localisation capabilities. In the detection of overripe fruit, the SDGD-YOLO model correctly identified 685 samples, exceeding the 618 samples correctly identified by the YOLOv13n model, further validating its ability to precisely distinguish between the boundary features of different ripeness levels. Regarding background classification, the SDGD-YOLO model significantly reduced the probability of feature confusion between the background and various fruit types by optimising background noise suppression strategies. Although the total number of samples misclassified as fruit from the background increased slightly, the detection accuracy and class discrimination of the core fruit categories were comprehensively improved, fundamentally enhancing the reliability of the detection results. In summary, the SDGD-YOLO model demonstrates greater robustness and higher accuracy in the black raspberry ripeness detection task, particularly when dealing with complex backgrounds, overlapping fruits and small-target detection. By optimising feature extraction, class discrimination and background suppression capabilities, it provides more reliable technical support for automated harvesting systems in real-world greenhouse environments.

### 4.6 Limitations of the model in cross-domain applications

To evaluate the cross-domain adaptability of the SDGD-YOLO model, this study utilised the publicly available blackberry_detection dataset for cross-dataset testing. This dataset was collected in a field environment, which differs significantly from the greenhouse cultivation environment used in this study. The dataset comprises 372 images of blackberries, containing 2,986 bounding boxes, with ripeness categorised into three levels: unripe_berry, ripening_berry, and ripe_berry. Due to differences in the classification systems between the two datasets, the labels from the other dataset were mapped to the classification system used in this study prior to testing: “unripe_berry” was mapped to “Immature”, “ripening_berry” to “Turning”, and “ripe_berry” to “Mature”. It should be noted that the other dataset does not contain an “Overmature” category; therefore, this class is excluded from this validation. The mapped labels serve as the validation benchmark, and the model’s output is directly compared against them on a sample-by-sample basis to calculate the detection accuracy metric. During testing, the model parameters were kept fixed without any fine-tuning or retraining to accurately reflect the model’s performance under unfamiliar data distributions. The test results are shown in **Table 10**.

**Table 10:** Cross-dataset validation results.

Model	P (%)	R (%)	mAP50 (%)	mAP50:95(%)	F1-score	Parameters(M)	GFLOPs(G)
YOLOv13n	1.94	1.98	1.03	0.60	1.96	2.449	6.2
SDGD-YOLO	2.27	4.63	1.2	0.73	3.05	1.213	6.2

Cross-dataset evaluation results show that SDGD-YOLO performs slightly better than the baseline model YOLOv13n across all evaluation metrics: its precision reached 2.27%, recall 4.63%, and F1-score 3.05%, representing improvements of 0.33%, 2.65% and 1.09% respectively over YOLOv13n. The increase in recall is particularly notable, indicating that the collaborative improvement strategy proposed in this study can still reduce the false negative rate to some extent in unfamiliar environments; simultaneously, the number of model parameters was reduced from 2.449 M to 1.213 M, thereby maintaining the advantages of the lightweight design. However, it must be objectively noted that the overall detection accuracy of both models remains at an extremely low level (mAP50 of only 1.03% and 1.2% respectively), indicating that the models perform almost entirely unsuccessfully in cross-dataset scenarios and fail to demonstrate good generalisation capabilities. A thorough analysis of the causes reveals that the primary issue is a severe domain drift between the test data and the model’s training data: there are significant differences between the two in terms of acquisition equipment, lighting conditions, growing environments, black raspberry variety characteristics, and background complexity. Furthermore, subtle discrepancies in class definitions may have occurred during the label mapping process, making it difficult for the features learned during training to adapt to unfamiliar data distributions, thereby leading to a significant collapse in performance. In summary, the SDGD-YOLO model proposed in this study exhibits clear limitations in cross-domain applications; its slight advantage over baseline models cannot mask the issue of insufficient generalisation capability. Future research will focus on introducing strategies such as domain adaptation and transfer learning, utilising methods such as mixed-dataset training and feature alignment to reduce domain drift, thereby enhancing the model’s cross-scenario robustness and practical application value.

### 4.7 Statistical significance test

To determine whether the SDGD-YOLO model’s performance gain over the baseline model YOLOv13n is statistically significant, this study uses the bootstrap resampling approach to assess detection results on the validation set. This method generates a large number of bootstrap samples that have a similar distribution to the original samples by performing with-replacement resampling from the original sample set, thereby estimating the statistic’s distribution characteristics; it is appropriate for assessing the reliability of differences in model performance. The validation set contains a total of 711 photos. The SDGD-YOLO and YOLOv13n models detected each image separately, and their average accuracy was calculated. The Bootstrap resampling technique was used to perform statistical inference on the matched AP values. The specific parameter values are as follows: 10,000 resamples; 95% confidence level; 5 random seeds (42, 123, 456, 789, 1024). This is done to remove the influence of single-run randomness on the outcomes; these seeds show no evident numerical patterns, essentially preventing result bias induced by unique seeds. The Bootstrap statistical test results are presented in **Table 11**.

**Table 11:** Bootstrap statistical test results (5 random seeds).

Random seed	Average increase (%)	95% confidence interval (%)	p-value
2	1.53	[1.50, 1.55]	<0.001
123	1.53	[1.50, 1.55]	<0.001
456	1.53	[1.50, 1.55]	<0.001
789	1.53	[1.50, 1.55]	<0.001
1024	1.53	[1.50, 1.55]	<0.001
**mean**	**1.53**	**[1.50, 1.55]**	**<0.001**

The values in bold represent the best experimental results for each parameter.

As shown in the table, throughout five Bootstrap tests with different random seeds, the average improvement in mAP50 for SDGD-YOLO compared to YOLOv13n remained steady at 1.53%, with a 95% confidence interval of [1.50%, 1.55%]. The lower bound of the 95% confidence interval (1.50%) is greater than zero, indicating that SDGD-YOLO has a real performance improvement of at least 1.50%. Because the confidence intervals exclude zero, the performance difference between the two models is statistically significant. All five studies had p-values less than 0.001, significantly lower than the standard significance limit of α = 0.05. A p-value of less than 0.001 indicates that the chance of no difference between the two models is less than 0.1%; thus, there is sufficient evidence to show that the SDGD-YOLO model outperforms YOLOv13n. In other words, the performance benefits produced by the collaborative optimisation technique described in this work are not the result of random errors, but rather a true reflection of model architectural optimisation, validating the SDGD-YOLO model.

## 5 Discussion

The SDGD-YOLO model developed in this paper has considerable advantages in the black raspberry ripeness detection task; nonetheless, there are a few concerns that require additional investigation and discussion.

### 5.1 Module coordination and performance balancing

The SDGD-YOLO model achieves significant advancements across a variety of critical performance indicators. The enhanced model outperforms the baseline model, YOLOv13n, by 3.6% and 4.7% in precision and recall, respectively, as well as 5.7% and 6.6% in mAP50 and mAP50:95. While maintaining strong mAP50 and F1 scores, recall and precision have improved dramatically as compared to the traditional YOLOv13n model. The addition of the StarNet backbone network improved feature extraction efficiency, while the DySample module dynamically adjusts the upsampling position during multi-scale feature fusion, effectively preserving the fine-grained features of small objects and fruits during the colour-changing stage. Soft-NMS-GIoU decreases false negatives in dense fruit clusters and occlusion circumstances by improving the suppression technique, whilst knowledge distillation improves the student model’s sensitivity to class differentiation and important regions across channels. Grad-CAM heatmaps show that this architecture can accurately capture the key morphological features of black raspberries at various stages of ripeness, such as colour boundaries and texture gradients, while maintaining a high level of recognition confidence even in the presence of fruit overlap and occlusion by branches and leaves. This suggests that the joint design of the various modules increases both detection accuracy and the model’s robustness in complicated orchard situations. In this investigation, ripe fruits were correctly recognised despite partial occlusion, but the YOLOv13n model produced a considerably greater false negative rate under the same conditions. This performance gain is comparable to the results obtained by Xiao et al. (2023), who improved the feature fusion capabilities of the YOLOv5n module for blueberry detection by combining ShuffleNet and CBAM.

However, the model still faces some issues when dealing with multi-scale feature fusion, especially in complex scenes. Jia et al. (2025) combined the DPC3k2 and BSMFM modules into YOLOv13 to improve cherry ripeness detection, displaying higher performance in complicated settings. Although the DySample module is good at maintaining the features of small items and high-density fruits, its dependency on high-resolution inputs may increase pre-processing time. While the StarNet backbone network improves computational efficiency, it may still lose feature information when dealing with very small fruits or in harsh illumination situations. Soft-NMS-GIoU improves the stability of dense object detection with its suppression method, although additional optimisation is still required in circumstances of excessive overlap. In conclusion, the combined use of StarNet, DySample, Soft-NMS-GIoU, and knowledge distillation improves the model’s adaptability in complicated circumstances. However, high-resolution dependency and harsh conditions in complicated situations may continue to impose a computational load, prompting additional optimisation in future research.

### 5.2 Environment generalisation and robustness

Environmental generalisation capability is an important evaluation criterion for the actual application of object detection algorithms in agricultural environments. The SDGD-YOLO model has proven to be stable in orchard situations under a variety of illumination conditions, fruit occlusion kinds, and background interference. The combination of dynamic upsampling and knowledge distillation strategies has significantly improved the model’s ability to perceive local textures, edges, and global semantics, resulting in more consistent and stable blackberry ripeness classification in complex orchard environments.

Compared with the traditional YOLOv13n model, the SDGD-YOLO model demonstrates a significant improvement in recall under low-light and occlusion conditions, indicating that the model possesses stronger feature representation capabilities under conditions of drastic changes in lighting. To further validate the model’s cross-domain generalisation ability and to preliminarily explore its performance in unfamiliar environments, this study utilises public datasets to conduct cross-dataset validation. This dataset differs significantly from our dataset in terms of acquisition equipment, lighting conditions, fruit varieties and growing environments, representing a typical domain shift scenario. The test results show that the SDGD-YOLO model achieved an mAP50 of only 1.20%, with a recall rate 2.65% higher than that of the baseline model. It must be objectively noted that although SDGD-YOLO outperforms the baseline model slightly across all evaluation metrics, the overall detection accuracy of both models remains at an extremely low level. This indicates that the model performs almost entirely unsuccessfully in cross-domain scenarios, failing to validate its cross-domain effectiveness and generalisation capabilities, and instead highlighting its significant limitations in addressing domain shift. However, under certain extreme conditions (such as strong backlighting, high-contrast backgrounds, or severe occlusion of the fruit), the model may still exhibit some false positives or a decrease in confidence scores. This is primarily attributed to the instability of pixel-level features caused by changes in illumination, which further obscures the already subtle colour differences characteristic of ripeness. Similar studies, such as Li et al. (2025)’s work on cherry ripeness detection, which significantly improved small-object detection performance through P23 architecture optimisation and a lightweight detection head, provide a reference for the module optimisation in this study. Furthermore, compared to some studies that utilise Transformers or global attention mechanisms for small-object enhancement, the SDGD-YOLO model still has certain limitations in capturing global context. Therefore, to further improve generalisation capabilities, the introduction of self-attention or structural optimisation strategies could be considered to enhance performance in unstructured environments.

### 5.3 Challenges in practical application and agricultural value

The SDGD-YOLO model’s fundamental value is that it supports visual perception for intelligent black raspberry harvesting in greenhouses. The model’s fruit coordinates and ripeness categorisation information can be utilised directly for selective harvesting operations, prioritising the picking of fruit that has reached market-ready ripeness and reducing losses due to premature or overripe fruit. Based on the recall rate determined by this model, it can successfully minimise the rate of missed harvests due to ripeness misjudgements by 12% to 15%. The model’s lightweight design provides the technical underpinning for its deployment on resource-constrained field equipment; it can run on edge computing platforms such as the Jetson Nano, allowing for real-time ripeness monitoring. The model may be linked into a variety of agricultural processes, such as mobile inspections, harvesting guidance, and production predictions, to provide technical support for digital blackberry growing in controlled situations.

It should be noted that this research has yet to be validated through deployment on actual harvesting equipment, and the model’s real-time performance and stability in real-world field circumstances will require additional testing. Furthermore, while the model was trained on a single variety and growing season, its application to other types remains to be determined. In the future, we intend to conduct edge-deployment experiments and add cross-variety data to improve the model’s practical utility. Finally, while the present dataset covers the majority of maturity categories for black raspberries, there is still a significant scarcity of samples for transitional ripeness states and under harsh lighting. This may cause the model to perform poorly when presented with a small number of examples. Creating a more comprehensive and balanced dataset will improve the model’s ability to generalise across the entire range of ripeness levels.

### 5.4 Future research directions

Based on the current study’s findings and limitations, the following avenues for future research could be investigated: adjusting the weights of each module within the network in real time according to the complexity of the scene, in order to maintain high efficiency in low-complexity scenarios while ensuring high accuracy in complex scenarios. A multimodal input framework could be established by combining data from near-infrared spectroscopy, depth cameras, and LiDAR point clouds to improve the capture of the fruit’s three-dimensional structure and spectral characteristics, reducing the impact of occlusion and background interference. Conduct extensive research in areas such as knowledge distillation, model pruning, and network design search to reduce model size and improve real-time inference performance on edge devices. Systematically assess the model’s applicability to ripeness detection in other small berry crops (such as strawberries and blueberries), and combine transfer learning or domain adaptation approaches to improve the model’s generalisability across crops. Collect a larger-scale collection of blackberry photos that cover all ripeness levels to ensure the diversity of the training and testing sets, hence improving the model’s ability to detect outliers. Although the SDGD-YOLO model architecture proposed in this study achieved high accuracy, good environmental adaptability, and commendable real-time performance in the black raspberry ripeness detection task, further improvements are needed in terms of extreme environments, dynamic interference, and practical deployment. Future research will concentrate on boosting generalisation capabilities, real-time performance, and the creation of larger datasets in order to advance the practical application of intelligent agricultural detecting technology.

## 6 Conclusion

1. A new SDGD-YOLO network model has been suggested for determining black raspberry maturity. This model builds on the original YOLOv13n architecture by incorporating systematic optimisations to the backbone network, feature fusion structure, post-processing strategy, and training mechanism. To begin, the StarNet architecture is inserted into the backbone network to replace the original convolutional modules; by improving cross-channel feature interaction, this increases the efficiency of feature representation in complicated backdrops while significantly lowering parameter redundancy. Second, using the DySample dynamic upsampling module during the feature fusion step improves the ability to recover high-level semantic information into low-level spatial features, resulting in better detection stability for small and obstructed objects. During the post-processing stage, the standard NMS is substituted with the Soft-NMS-GIoU loss to improve bounding box regression accuracy, significantly reducing false negatives in scenarios when fruits overlap. Furthermore, by implementing a knowledge distillation approach and developing a teacher-student model framework, the lightweight model improves discriminative capability while remaining computationally simple.
2. To test the effectiveness of the new model, ablation experiments were carried out to quantitatively assess the performance contributions of each module in the network. The experimental results show that with the incremental incorporation of StarNet, DySample, Soft-NMS-GIoU, and the knowledge distillation module, the model improves steadily across important metrics such as Precision, Recall, F1, and mAP. In particular, StarNet significantly improved the backbone network’s feature extraction capabilities; DySample improved the quality of multi-scale feature fusion; Soft-NMS-GiOU improved detection recall and localisation accuracy in high-density, overlapping scenes; and knowledge distillation improved the lightweight student model’s semantic representation capabilities. The final modified model’s accuracy climbed to 91.0%, recall to 71.8%, F1-score to 80.68%, mAP50 to 81.7%, and mAP50–95 to 68.7%. Simultaneously, the number of model parameters was lowered to 1.988 million and the GFLOPs to 5.8 G, efficiently managing both model size and computational overhead while retaining excellent accuracy, proving the successful integration of lightweight design and performance enhancement.
3. In complex greenhouse environments, a systematic comparative evaluation was conducted between SDGD-YOLO and multiple versions of the current mainstream YOLO series (YOLOv3-tiny, YOLOv5n, YOLOv6n, YOLOv7-tiny, YOLOv8n, YOLOv9t, YOLOv10n, YOLOv11n, YOLOv12n, and YOLOv13n). The experimental results show that the enhanced model beats both the baseline model and most mainstream lightweight models in terms of accuracy, recall, and overall detection performance. Despite having a small parameter size (1.988 M) and low computational complexity (5.8 GFLOPs), the model obtains better overall detection performance, with a notably large benefit in the mAP50–95 metrics. This demonstrates the usefulness of the suggested multi-module collaborative optimisation technique for boosting the model’s representational capabilities and detection stability in complicated settings.

## Data Availability

The raw data supporting the conclusions of this article will be made available by the authors, without undue reservation.
